# CARD-FISH in the Sequencing Era: Opening a New Universe of Protistan Ecology

**DOI:** 10.3389/fmicb.2021.640066

**Published:** 2021-03-04

**Authors:** Kasia Piwosz, Indranil Mukherjee, Michaela M. Salcher, Vesna Grujčić, Karel Šimek

**Affiliations:** ^1^Department of Fisheries Oceanography and Marine Ecology, National Marine Fisheries Research Institute, Gdynia, Poland; ^2^Centre ALGATECH, Institute of Microbiology of the Czech Academy of Sciences, Třeboň, Czechia; ^3^Biology Centre of the Czech Academy of Sciences, Institute of Hydrobiology, České Budějovice, Czechia; ^4^Science for Life Laboratory, Department of Gene Technology, School of Engineering Sciences in Chemistry, Biotechnology and Health, KTH Royal Institute of Technology, Stockholm, Sweden

**Keywords:** aquatic microbial food webs, CARD-FISH, grazing by protists, protists, unicellular eukaryotes, bacterivorous, omnivorous and predatory flagellates, heterotrophic and mixotrophic flagellates

## Abstract

Phagotrophic protists are key players in aquatic food webs. Although sequencing-based studies have revealed their enormous diversity, ecological information on *in situ* abundance, feeding modes, grazing preferences, and growth rates of specific lineages can be reliably obtained only using microscopy-based molecular methods, such as Catalyzed Reporter Deposition-Fluorescence *in situ* Hybridization (CARD-FISH). CARD-FISH is commonly applied to study prokaryotes, but less so to microbial eukaryotes. Application of this technique revealed that *Paraphysomonas* or *Spumella*-like chrysophytes, considered to be among the most prominent members of protistan communities in pelagic environments, are omnipresent but actually less abundant than expected, in contrast to little known groups such as heterotrophic cryptophyte lineages (e.g., CRY1), cercozoans, katablepharids, or the MAST lineages. Combination of CARD-FISH with tracer techniques and application of double CARD-FISH allow visualization of food vacuole contents of specific flagellate groups, thus considerably challenging our current, simplistic view that they are predominantly bacterivores. Experimental manipulations with natural communities revealed that larger flagellates are actually omnivores ingesting both prokaryotes and other protists. These new findings justify our proposition of an updated model of microbial food webs in pelagic environments, reflecting more authentically the complex trophic interactions and specific roles of flagellated protists, with inclusion of at least two additional trophic levels in the nanoplankton size fraction. Moreover, we provide a detailed CARD-FISH protocol for protists, exemplified on mixo- and heterotrophic nanoplanktonic flagellates, together with tips on probe design, a troubleshooting guide addressing most frequent obstacles, and an exhaustive list of published probes targeting protists.

## Introduction

Protists (unicellular eukaryotes) play a central role in carbon and energy flow and nutrient recycling in aquatic food webs (Azam et al., 1983; Sherr and Sherr, 1988). Small heterotrophic and mixotrophic flagellated (HF and MF, respectively) protists with cell size < 8 μm are the key predators of pelagic prokaryotes (Porter et al., 1985; Šimek and Straškrabová, 1992; Rychert, 2006; Zubkov and Tarran, 2008). They control size distribution and composition of prokaryotic communities by selectively grazing on specific morpho- and phylotypes (Šimek and Chrzanowski, 1992; Šimek et al., 1997b; Jürgens and Matz, 2002; Jezbera et al., 2005, 2006). The trophic role of larger protists (8–20 μm) is far less understood (Arndt and Mathes, 1991; Arndt et al., 2000; Šimek et al., 2020). These apparently omnivorous predators appear ahead of the ciliates in the energy transfer and are the main consumers of bacterivorous protists and pico-sized algae (Piwosz and Pernthaler, 2011; Mukherjee et al., 2017, 2019; Grujčić et al., 2018). These considerably understudied but ecologically highly relevant trophic interactions significantly modulate carbon flow efficiency, nutrient regeneration, and overall trophic cascading within the grazer food chain (Goldman and Caron, 1985; Goldman et al., 1985; Andersen et al., 1986; Arndt et al., 2000; Šimek et al., 2019, 2020). Our review focuses on phagotrophic nanoplanktonic HF and MF (with cell size 3–20 μm; Jürgens and Matz, 2002; Massana, 2011), whose grazing impacts modulate their prey communities, and *vice versa*, shifts in prokaryotic prey communities rapidly cascade to the predator communities (Šimek et al., 2013, 2020; Grujčić et al., 2018), while our knowledge on these key predator-prey interactions at an individual level is quite limited.

Here, we review how the application of the CARD-FISH technique helped to advance our understanding of microbial trophic interactions and energy fluxes through microbial food webs, with the focus on aquatic nanoplanktonic flagellates (NF). First, we shortly discuss the currently used CARD-FISH protocols for protists and their combinations with other fluorescence-labeling techniques that allow determination of feeding modes and preferred prey at a single cell level in natural environments without any sample manipulation. Subsequently, we demonstrate how these approaches revealed hidden ecological and ecophysiological traits of so far unknown and morphologically almost indistinguishable protistan lineages in marine and freshwater habitats. Based on this novel knowledge that considerably modifies the current views on the carbon fluxes in pelagic environments, we propose an updated model of microbial food webs that more realistically reflects the complex trophic interactions and specific roles of NF. Our overarching aim is to attract more attention of microbial ecologists to the intriguing aspects of protistan ecology that these single-cell resolution approaches enabled to discover, thus considerably advancing our understanding of microbial food web structure and functioning.

## *Infusoria*: ‘Black Box’ of Protists

The existence of protists was discovered in the 17th century by Antonie van Leeuwenhoek, who was the inventor of a light microscope, the main tool used in ecological studies of these captivating organisms until the late 20th century. This technique provided basic information on the morphology and gave preliminary hints on feeding modes of the most commonly observed and cultured microplanktonic protists (cell size > 20 μm) that possess diverse cell structures and shapes allowing for tentative discrimination of species. Thus, ciliates or dinoflagellates can be microscopically classified, and a large number of planktonic taxa have been described (Foissner and Berger, 1996; Steidinger and Jangen, 1997; Foissner et al., 1999; Hoppenrath et al., 2009, 2014; Madoni, 2011). Consequently, in ecological studies of ciliates, feeding modes of the major bacterivorous and omnivorous taxa can be identified to the level of genus, species or of a genus-like morphotype in environmental samples (e.g., Montagnes and Lynn, 1987; Šimek et al., 2000, 2019; Posch et al., 2015; Šimek and Sirova, 2019).

In contrast, it is far harder to differentiate NF to a species, genus, or even phylum level. The research on their community composition and ecological traits has been largely hindered by virtual lack of distinguishable morphological features of these mostly uncultivated protists (Arndt et al., 2000; Bass and Cavalier-Smith, 2004; Adl et al., 2019). Their enormous phylogenetic diversity is largely hidden behind simple, oval cells usually containing a single nucleus and 1–2 flagella. Only a few NF groups, e.g., cryptophytes, pedinellids, haptophytes, kinetoplastids and choanoflagellates, can be identified by experts via phase contrast, fluorescence microscopy, electron microscopy, or live sample observations (Swale, 1969; Patterson and Larsen, 1991; Boenigk and Arndt, 2002; Sekiguchi et al., 2003; Wasmund et al., 2011; Majaneva et al., 2012; Jeuck and Arndt, 2013; Weber et al., 2017). Consequently, in classical grazing research on heterotrophic prokaryotes, bacterivorous NF were treated as one functional unit, which reacts more or less uniformly to certain environmental factors (Berninger et al., 1991; Sanders et al., 1992; Gasol and Vagué, 1993). Although such microscopy-based studies reported, for example, similar looking small HF with two unequally long flagella as “*Spumella*-like,” “(*Pseudo)Bodo* ssp.,” or “*Paraphysomonas* spp.” functional guilds of key bacterivores in marine and freshwaters, they failed to uncover their phylogenetic affiliation and diversity (Fenchel, 1982; Andersen and Fenchel, 1985; Jürgens and Güde, 1994; Hansen, 1996; Šimek et al., 1997a; Matz et al., 2002). Nevertheless, epifluorescence microscopy, combined with the use of fluorescent food tracers or radiolabeled prey, allowed to recognize smaller flagellated protists (ca. 2–8 μm in size) as the most prominent pelagic bacterivorous protistan groups, omnipresent in both marine and freshwater habitats (Sherr and Sherr, 1988; Chrzanowski and Šimek, 1990; González et al., 1990b; Nygaard and Hessen, 1990; Berninger et al., 1991; Šimek et al., 1997b; Zubkov et al., 1998; Jezbera et al., 2005; Weisse et al., 2016). Moreover, even this simplifying “black box approach,” ranking HF and prokaryotes only as large functional guilds, has brought many indications that bacterioplankton responds to strong HF grazing pressure by multitude of adaptive mechanisms. For instance, selective grazing of HF predators shape morphological and compositional structure of prokaryotes (Chrzanowski and Šimek, 1990; González et al., 1990a; Šimek and Chrzanowski, 1992; del Giorgio et al., 1996; Corno and Jürgens, 2006; Zubkov and Tarran, 2008), resulting in a broad variety of grazing-resistant strategies of prokaryotes (see e.g., reviews by Hahn and Höfle, 2001; Jürgens and Matz, 2002; Pernthaler, 2005) that lead to complete avoidance or a considerable decrease of their grazing-induced mortality rates (Jürgens and Güde, 1994; Šimek et al., 1999, 2001; Langenheder and Jürgens, 2001). However, only the application of FISH techniques to target specific lineages of prokaryotes brought detailed insights to diverse impacts of protistan grazing on prokaryotic morphology and community composition (Šimek et al., 1997b, 2001, 2007; Langenheder and Jürgens, 2001; Jürgens and Matz, 2002; Pernthaler, 2005).

Cultivation approaches and live sample observations (e.g., Arndt et al., 2000; Boenigk and Arndt, 2002; Jeuck and Arndt, 2013; Weber et al., 2017) provided additional details for protists determination and thus also facilitated progress in the field, but have been limited to a restricted number of easily cultivable flagellates and ciliates. Unfortunately, the vast majority of free-living protists cannot be easily isolated and cultivated under close to *in situ* conditions (Weber et al., 2017), and thus, in fact this bottleneck effect recalls the well-known bacterial story of “great plate count anomaly” (Staley and Konopka, 1985; Lim et al., 1999).

## *Sequencing Era*: Opening the “Black Box” of Protists

**FIGURE 1:**
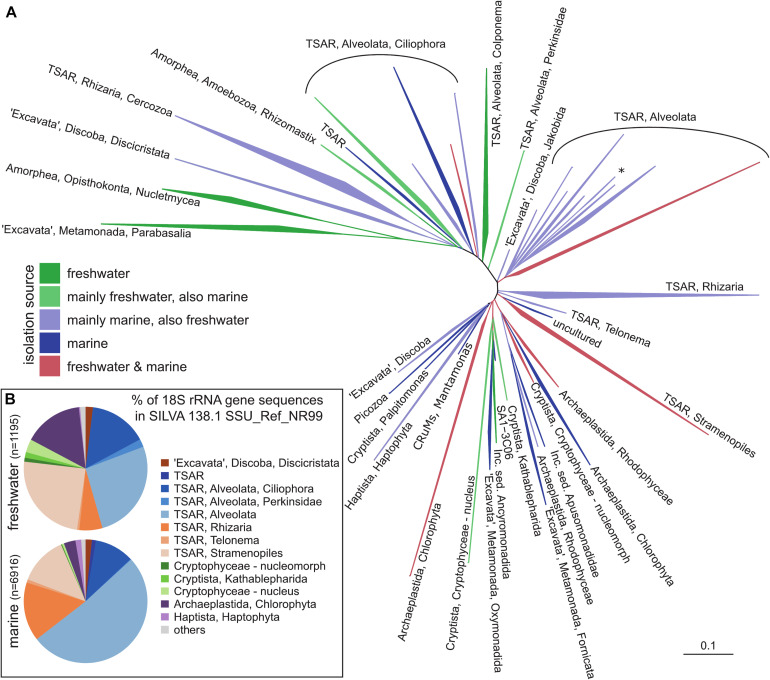
A general phylogenetic tree of protistan groups known to be important in pelagic environments. **(A)** 18S rRNA phylogenetic guide tree provided by SILVA release 138.1 SSU_Ref_NR99. Only branches containing sequences recovered either from freshwater and marine habitats were included by parsing the isolation_source field for respective entries, higher organisms (plants, animals) have been removed. **(B)** Taxonomic classification of eukaryotic 18S rRNA gene sequences recovered either from freshwater or marine habitats. The names of the groups follow notation by Adl et al. (2019) and Burki et al. (2020). TSAR – telonemids, stramenopiles, alveolates, and Rhizaria; CRuMs – collodictyonids (syn. diphylleids), Rigifilida and *Mantamonas*. The asterisk in TSAR, Alveolata indicates a lineage of non-TSAR protists (*Archaeplastida, Rhodophyceae, Galdieria*).

The vast diversity of protists that encompass all the branches of eukaryotic phylogenetic tree (Figure 1A) has been recognized only since the last two decades (Pawlowski et al., 2012; Adl et al., 2019; Burki et al., 2020). The progress in describing the diversity of nanoplanktonic protists has been possible thanks to the development of molecular techniques and their increased application in microbial ecology. The increasing amount of information about their phylogenetic diversity, discoveries of cryptic species and of novel lineages made it evident that the knowledge on their diversity had been superficial (Šlapeta et al., 2006; Caron et al., 2012). These unexpected results have changed not only our perception of protistan diversity, but also of their role in ecosystem functioning and evolution of eukaryotes (Worden et al., 2015; Adl et al., 2019; Burki et al., 2020).

As in the case of prokaryotes, a gene encoding the small subunit (SSU) ribosomal RNA (18S rRNA) became widely used as a marker gene in community analyses of protists (Figure 1A). Due to its patchy nature of conserved and non-conserved regions, it enables both discrimination between closely related organisms and primer design for specific groups at various taxonomical levels. The first studies by Sanger sequencing of 18S rRNA clone libraries revealed an unexpected diversity and presence of novel lineages of protists from marine ecosystems (Lopez-Garcia et al., 2001; Moon-van der Staay et al., 2001). A wide range of studies followed in various ecosystems such as marine, freshwaters, sediments, soils, and insect guts, to name a few (Edgcomb et al., 2002; López-García et al., 2003; Romari and Vaulot, 2004; Lovejoy et al., 2006; Moon-van der Staay et al., 2006; Stoeck et al., 2006; Ohkuma and Brune, 2010; Piwosz and Pernthaler, 2010; Sauvadet et al., 2010; Scheckenbach et al., 2010; Stock et al., 2012; Medlin et al., 2017; Mukherjee et al., 2017). With the advent of sequencing technologies, Sanger sequencing of clone libraries has been replaced by high-throughput sequencing (HTS, also known as NGS – Next generation sequencing) techniques, such as already obsolete Ion torrent and 454 pyrosequencing (Behnke et al., 2011; Bachy et al., 2013; Egge et al., 2013, 2015; Georges et al., 2014; Balzano et al., 2015; Piwosz et al., 2018), currently the most often used Illumina MiSeq/HiSeq platforms (Logares et al., 2014; de Vargas et al., 2015; Hu et al., 2016), and the most recent PacBio and Oxford Nanopore MinION platforms (Orr et al., 2018; Davidov et al., 2020; Hatfield et al., 2020). HTS enables to process large number of samples at once at low cost, providing qualitative information on composition of protistan communities even up to the species level (Amaral-Zettler et al., 2009; Stoeck et al., 2010; Logares et al., 2014). The Malaspina (Duarte, 2015) and TARA Oceans (Bork et al., 2015) oceanographic circumnavigation campaigns made it evident that the majority of marine protistan lineages remain morphologically unknown, even if they belong to the recognized groups, such as Stramenopiles, Alveolata, Rhizaria, and “Excavata” (Figure 1B, de Vargas et al., 2015; Pernice et al., 2016). Similar pictures also emerged from localized sampling campaigns (Parris et al., 2014; Massana et al., 2015). Highly diverse communities of Dinophyta, Cercozoa, Stramenopiles, and Kinetoplastida were reported from extreme environments like abyssal depths and hydrothermal vents (Sauvadet et al., 2010; Scheckenbach et al., 2010). A novel picture on protistan diversity also emerged from the studies of freshwater and brackish habitats (Figure 1B), which discovered an unexpected diversity of the well-known groups: Cryptophyta, Stramenopiles, and Choanozoa (del Campo and Massana, 2011; Simon et al., 2015a, b; Grossmann et al., 2016; Hu et al., 2016; Grujčić et al., 2018; Piwosz et al., 2018). One of the biggest surprises from these studies was the discovery of highly diverse marine lineages of diplonemids (Flegontova et al., 2016), with a recently discovered lineage also in freshwaters (Mukherjee et al., 2020). Similarly, another member of the excavate protists, kinetoplastids, were reported to be highly diverse and abundant in the deep waters of both oceans and freshwater lakes (von der Heyden and Cavalier-Smith, 2005; Scheckenbach et al., 2010; Salani et al., 2011; Flegontova et al., 2018; Mukherjee et al., 2019). Novel lineages were discovered within well-known groups, such as the freshwater CRY1 lineage of cryptophytes (Shalchian-Tabrizi et al., 2008). Finally, Perkinsozoa (Mangot et al., 2012; Taib et al., 2013) and *Telonemia* were also found to be widely distributed in both marine and freshwaters (Shalchian-Tabrizi et al., 2007; Brate et al., 2010; Triado-Margarit and Casamayor, 2012; Simon et al., 2015b). However, the ecological role of most of these novel groups is not yet well understood.

To circumvent this lack of ecophysiological information, HTS sequencing studies have been combined with advanced statistical analyses, e.g., multivariate or co-occurrence networks (Steele et al., 2011; Cram et al., 2013; Guidi et al., 2016). Such correlative approaches provide only indirect hints about the biotic and abiotic interactions that may be crucial for structuring microbial communities over time and space (Posch et al., 2015; Qu et al., 2021), but may enable informed decision on the microbial groups to be targeted by CARD-FISH. However, the relative ease of obtaining sequencing data from large amounts of samples at once compared to analyzing individual samples by laborious microscopic methods resulted in a disproportional boom in diversity research. Unfortunately, the rapid progress in sequence-based investigation has not been accompanied by an increase in studies on other aspects of protistan ecology, such as morphology, abundances, feeding modes, or trophic roles of new lineages (Stern et al., 2018). Most strikingly, for most recovered sequences, neither the organisms themselves have been visualized yet nor their abundance and distribution have been quantified. In addition, much more efforts have been put on prokaryotes than on protists, although the latter play key roles as primary producers, predators or parasites. This evident imbalance in the research progress represents likely one of the largest knowledge gaps in the field, which justifies also the timing of this review.

**FIGURE 2:**
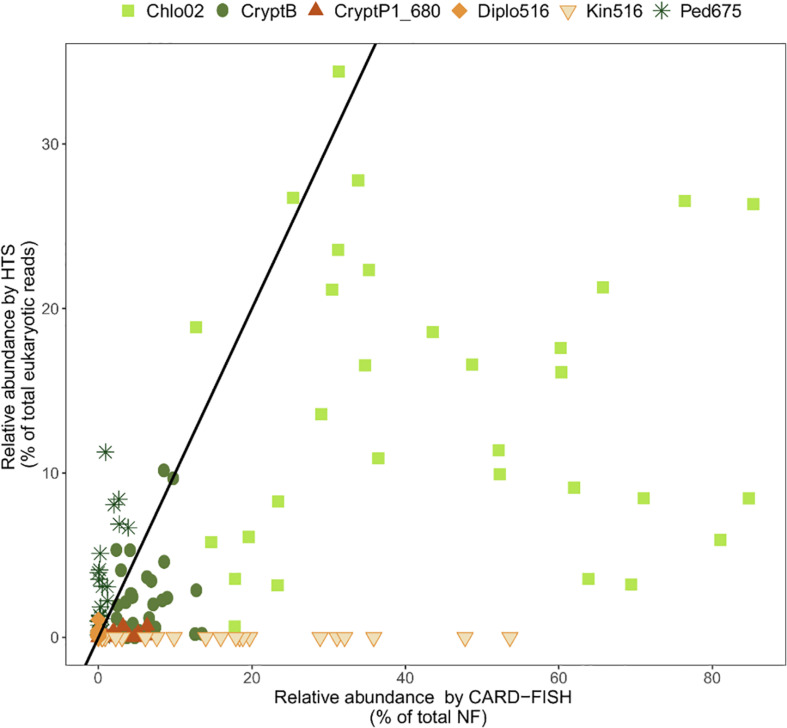
Relationships between relative abundance of selected protistan lineages from high throughput sequencing (HTS) and CARD-FISH. Black line indicates a perfect 1:1 correspondence between the two methods. Points above this line indicate overrepresentation, and below the line underrepresentation of a phylotype by HTS. Chlo2, chlorophytes hybridizable with Chl02 probe; CryptB, cryptophytes hybridizable with CryptB; CryptP1_680, CRY1 cryptophytes hybridizable with CryptP1_680 probe; Diplo516, diplonemids hybridizable with Diplo516 probe; Kin516, kinetoplastids hybridizable with Kin516 probe; Ped675, pedinellids hybridizable with Ped675 probe. Detail on the probes can be found in Supplementary Table 1. Data on Chlo02, CryptB, CryptP1_680 and Ped675 are from Piwosz et al. (2020), on Diplo516 from Mukherjee et al. (2020), and on Kin516 from Mukherjee et al. (2019).

Moreover, the quantitative accuracy of HTS data is compromised by the uneven effectiveness of DNA extraction protocols (Martin-Laurent et al., 2001), biased efficiency of PCR amplification (Hansen et al., 1998), incomplete coverage of primers across phylogenetic groups (Klindworth et al., 2013; Mukherjee et al., 2015), and a highly variable number of 18S rDNA gene copies in protists ranging from 1 in picoplanktonic *Nannochloropsis salina* (Eustigmatophyceae) to 315,000 in the peritrich ciliate *Vorticella* sp. (Zhu et al., 2005; Gong et al., 2013). Moreover, high intra-genomic diversity of 18S rRNA gene sequences (i.e., the same cell can harbor multiple variants of 18S rRNA genes beyond the species level) can artificially inflate the diversity of some protistan groups (Caron and Hu, 2019; Mukherjee et al., 2020). The relative abundances of specific protistan phylotypes obtained by HTS poorly correspond to their relative abundances in the original samples (Figure 2), and provide only a limited possibility to conduct hypothesis-driven research on the ecology of particular protistan taxa (Pitsch et al., 2019; Piwosz et al., 2020). In results, little is known about the ecology of majority of the newly discovered protistan lineages. Even the latest single cell sequencing or metagenomic techniques cannot yet provide ecological data on uncultured protistan lineages due to technological obstacles and the knowledge gaps in eukaryotic genomics (Seeleuthner et al., 2018).

## Catalyzed Reporter Deposition—Fluorescence *in situ* Hybridization: Opening a New Universe of Protistan Ecology

**FIGURE 3:**
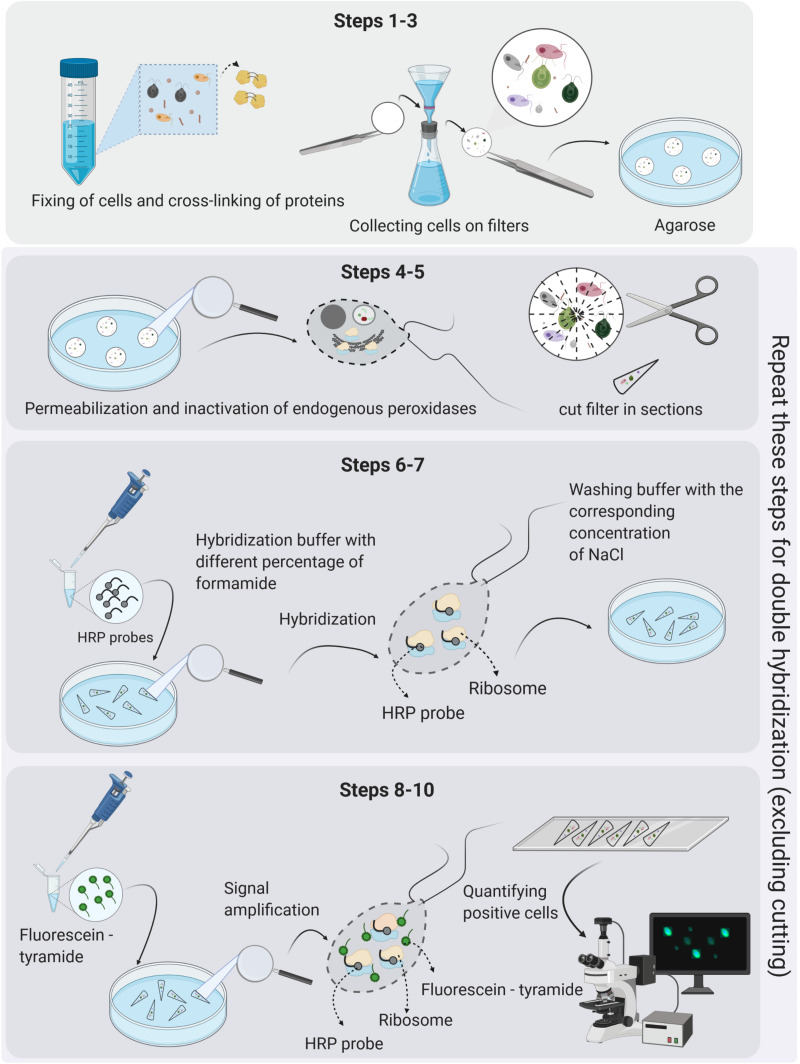
The key steps in the CARD-FISH and double CARD-FISH procedures. The detailed protocol is given in Supplementary File 2, and a single page printable version in Supplementary File 3. Figure created with BioRender.

The hindrances caused due to the sole use of sequencing techniques to address ecological questions can be solved by complementary use of microscopy-based molecular methods, such as fluorescence *in situ* hybridization (FISH) (Amann et al., 1995). FISH and its improved version with enzymatic signal amplification (catalyzed reporter deposition “CARD-FISH,” known also as tyramide signal amplification “TSA-FISH”), provide estimates of relative abundance (a percent contribution to total eukaryotic numbers) of individual microbial lineages defined by their rRNA gene phylogeny (Not et al., 2002; Pernthaler et al., 2004). The key steps in the CARD-FISH protocol are depicted in Figure 3. CARD-FISH provides up to 200-fold brighter signals than FISH with monolabeled probes, enabling detection of almost inactive cells with a low number of ribosomes (Lim et al., 1993). It has become a verified quantitative tool in numerous studies on prokaryotic communities, providing hints on their ecological niche, functions, and interactions (Amann and Fuchs, 2008). However, its use for eukaryotes has been less common (e.g., Not et al., 2005, 2008; Massana et al., 2006a; Piwosz and Pernthaler, 2010; Unrein et al., 2014; Mukherjee et al., 2015). The main advantage of CARD-FISH over the HTS methods is that the relative abundance of a particular lineage can be evaluated independently from all other taxa in the samples. Moreover, the CARD-FISH procedure can be separately optimized for each target group (probe), which is not possible for PCR with primers that target many different templates. It can be also combined with results from direct enumeration methods, such as microscopy or flow cytometry, providing absolute abundance estimates of microbial lineages in the samples. Finally, simultaneous use of CARD-FISH and double CARD-FISH with fluorescent tracers and food vacuole content observations provides information on grazing rates, prey-selectivity, taxonomic information on bacterial, archaeal and eukaryotic prey, and ultimately trophic roles of specific protistan lineages (Jezbera et al., 2005; Chambouvet et al., 2008; Piwosz and Pernthaler, 2010, 2011; Ballen-Segura et al., 2017; Grujčić et al., 2018; Šimek et al., 2020). Double CARD-FISH is a powerful tool for examining bacterivory and omnivory by combining two probes at different trophic levels, targeting protistan predators as well as their prey in food vacuoles (both prokaryotes or other protists). This combination gives new insights into predator–prey interactions, directly demonstrating which bacteria or small protists are preferentially consumed and which groups of flagellates are their grazers in aquatic ecosystems (Piwosz and Pernthaler, 2010, 2011; Grujčić et al., 2018). Further, this method can be applied to identify symbiotic relationships between pro- and eukaryotic microbes (Chambouvet et al., 2008; Dirren et al., 2014; Lepère et al., 2016).

**FIGURE 4:**
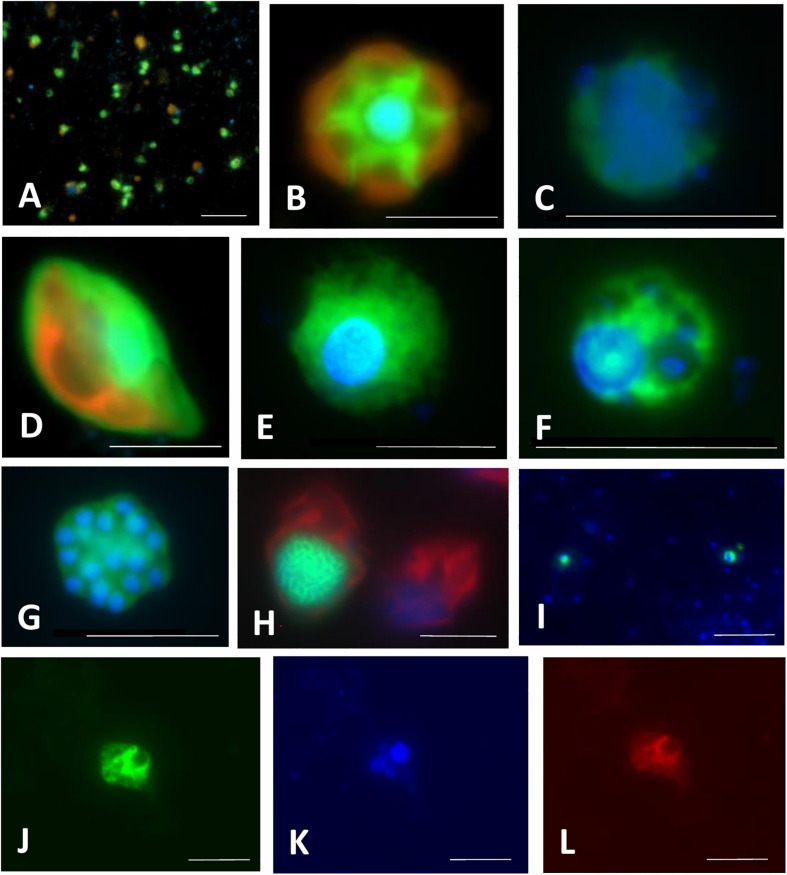
Microphotographs showing typical flagellate size and morphology of cells targeted by CARD-FISH probes from brackish waters of the Baltic Sea **(A–H)** and freshwater lakes **(I–L)**. Shown are overlay images of DAPI-stained flagellate nuclei (blue), FITC or Alexa488 stained flagellates targeted by probes (green) and chlorophyll autofluorescence (orange/red), except for **(J–L)**, where individual pictures of different channels are shown. **(A)** Picoplanktonic chlorophytes (probe Chlo02) and other unhybridized algae; **(B)** plastidic pedinellid (probe Ped675); **(C)** aplastidic pedinellid (probe Ped675); **(D)** plastidic cryptophyte (probe Crypto B); **(E,F)** large and small morphotypes of MAST-6 stramenopiles, respectively (probe MAST-6); **(G)** parasitic Syndiniales (probe Alv_Bal02): a trophont outside a host, **(H)** parasitic Syndiniales (probe Alv_Bal02): a trophont inside a host cell (the dinoflagellate *Heterocapsa triquetra*); **(I)** tiny perkinsozoans (probe PERKIN_01); **(J–L)** a diplonemid cell hybridized with probe DiploR1792 (green) **(J)**; corresponding DAPI staining (blue) **(K)** and double CARD-FISH with general eukaryotic probe [Euk1209, Alexa546 (red), **L**]. Scale bar = 10 μm.

**FIGURE 5:**
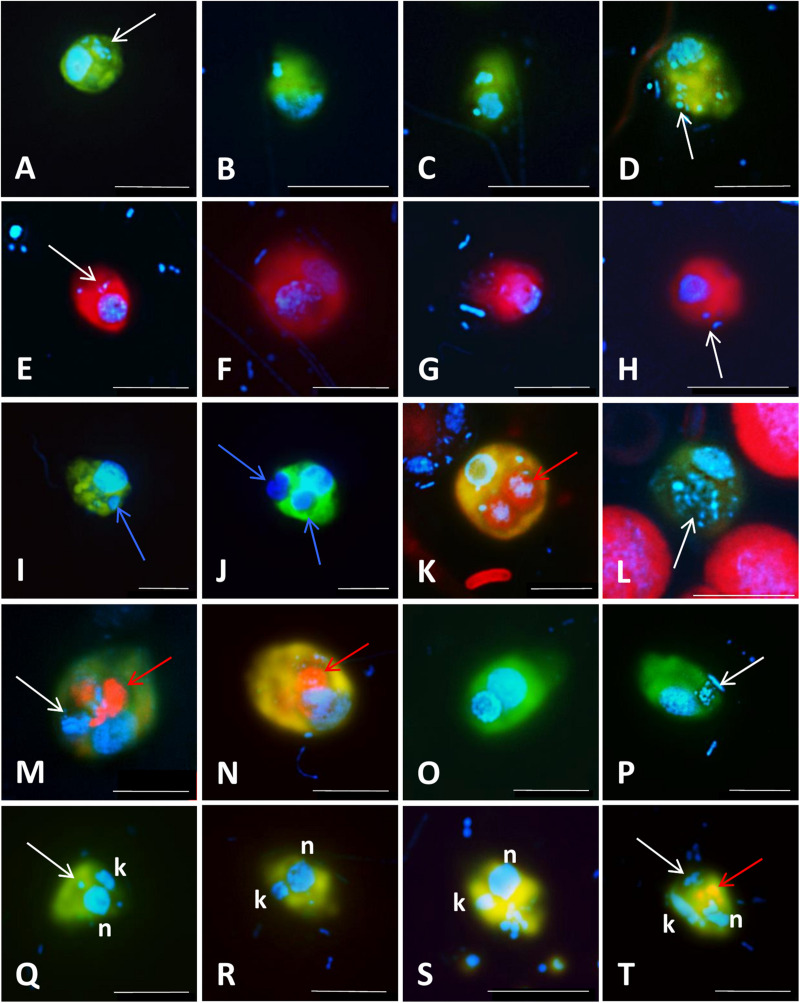
Microphotographs showing food preferences, i.e., bacterivory **(A–H,M,P–T)**, predatory **(I,J)**, or omnivory **(M,N,P)** of freshwater flagellates from different eutrophic ponds **(A–D,I–T)** and from mesotrophic Římov reservoir **(E–H)** in Czech Republic. Shown are overlay Z-stack images (following the methods described in Šimek et al., 2019) of flagellates targeted by probes [**A–D**,**I–T:** FITC-stained flagellates (yellow); **E–H:** Alexa546-stained flagellates (red)], DAPI-stained bacteria and flagellate nuclei (blue) and autofluorescence of algae and cyanobacteria (red). **(A–D)** Heterotrophic lineage of Cryptophyta (probe Cry1-652) with ingested bacteria; **(E–H)** other Cryptophyta (general probe Crypto B) with ingested bacteria; **(I,J)** Cercozoans of Novel Clade 7 (probe Cerc-193) with ingested flagellate prey or their cell remains; **(K,L)** other Cercozoa (general probe Cerc-02) with ingested cyanobacterial cells of *Microcystis* **(K)** or bacteria **(L)**; **(M–P)** Katablepharidacea (probe Kat-1452) with ingested algal **(M,N)** or bacterial prey **(P)**; **(Q–T)** kinetoplastids (probe Kin516) with visible DAPI-stained nucleus **(n)**, kinetoplast **(k),** and ingested bacteria. White, blue, and red arrows highlight examples of ingested bacteria **(A–H,L,M,P,Q,T)**, flagellate prey **(I,J)** and algae and cyanobacteria **(K,M,N,T)**, respectively, visible in the grazer food vacuoles. Two arrows (images **M,N,T**) point to parallel appearance of algae and bacteria in food vacuoles of the same flagellate cells, thus indicating omnivory of the grazer. The scale bar shows length of 5 μm in all images except for **(F,I,J,M)**, with the scale bar showing 10 μm.

**FIGURE 6:**
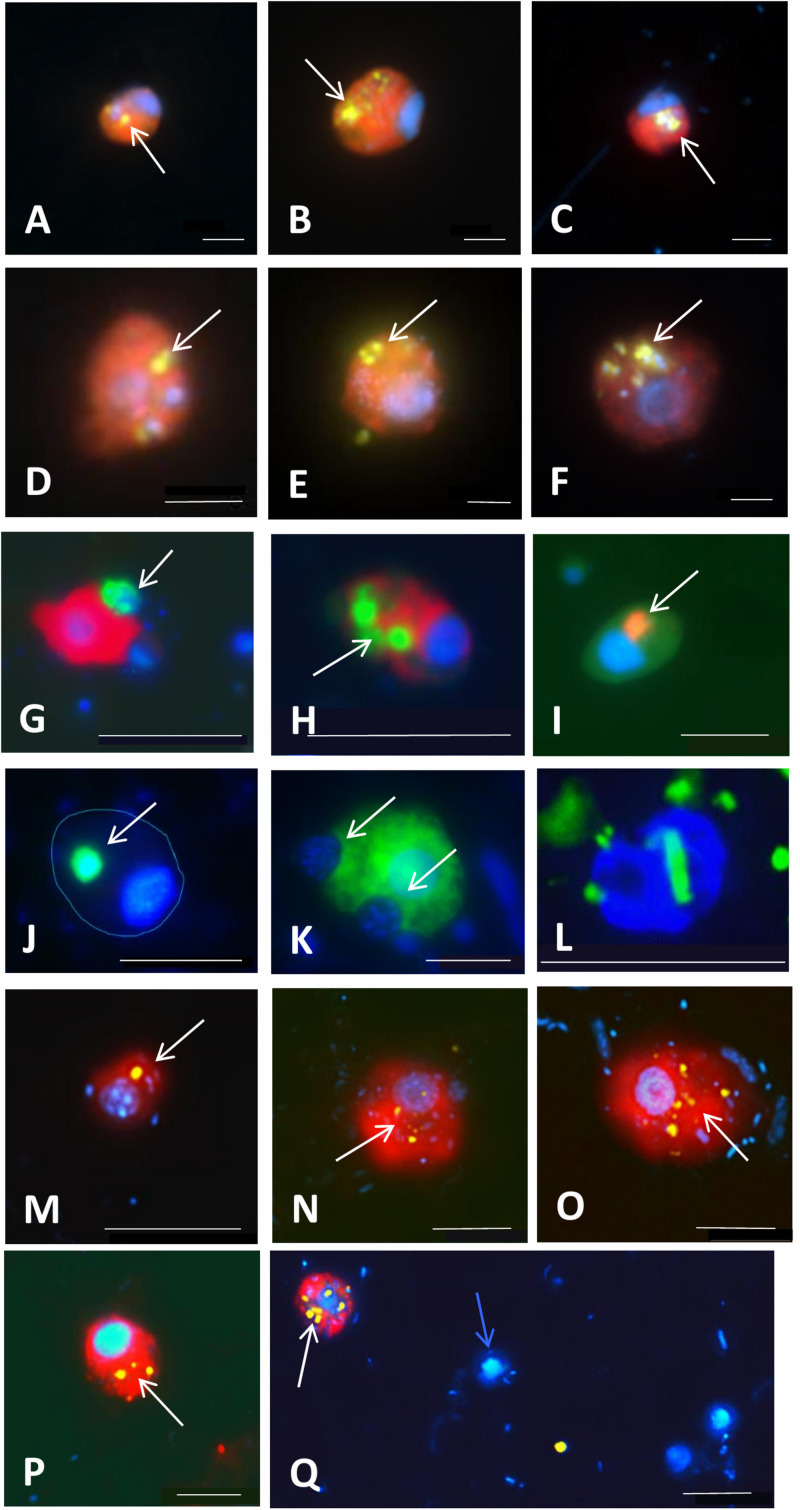
Examples of application of double CARD-FISH **(A–H)**, food vacuole content observations **(I–K)**, or a combination of CARD-FISH and uptake of fluorescently labeled bacteria (FLB; **L–Q**) to examine food preferences or bacterial uptake rates in freshwater and brackish environments. Double CARD-FISH of **(A–C)** aplastidic CRY1 lineage of Cryptophyta [probe Cry1-652, (red)]; and **(D–F)** aplastidic Cryptophyta [probe Crypto B, (red)] feeding on various Betaproteobacteria (yellow) (Grujčić et al., 2018); **(G)** Cercozoa [probe Bal_02, (red)] feeding on other Cercozoa [probe Bal_01, (green)]; **(H)** Cercozoa [probe Bal_02, (red)] feeding on prymnesiophytes [probe Prym02, (green)]; **(I)** Cercozoa [probe Bal_02, (red)] grazed by an unidentified protist; **(J)** MAST-6 stramenopile [probe MAST-6, (green)] grazed by an unidentified protist (cell shape indicated with blue line); **(K)** MAST-6 stramenopile [probe MAST-6, (green)] engulfing algae. Each image is an overlay of three pictures of the same flagellate cell observed under ultraviolet excitation (showing the blue nucleus after DAPI-staining), green light excitation (red color corresponding to different flagellate groups labeled with Alexa546 using CARD-FISH) and blue light excitation (yellow-green color corresponding to ingested Betaproteobacteria labeled with FITC using CARD-FISH and green color corresponding to ingested protists labeled with Alexa488 using CARD-FISH). Grazing on FLB [yellow] by **(L)** Cercozoa [probe Bal_01, labeled with Alexa350 (blue)]; and aplastidic cryptophytes [probe Crypto B, (red)] **(N–Q)** and CRY1 lineage of Cryptophyta [probe Cry1-652, (red)] **(M)**]. White arrows indicate ingested prokaryotes and eukaryotes targeted by CARD-FISH probes or FLB in food vacuoles of grazer cells. A blue arrow indicates a non-target flagellate cell close to a Cry1-positive cell with ingested FLB **(Q)**. Scale bar = 2 μm **(A–F)**, 10 μm **(G–L)**, and 5 μm **(M–Q)**.

**FIGURE 7:**
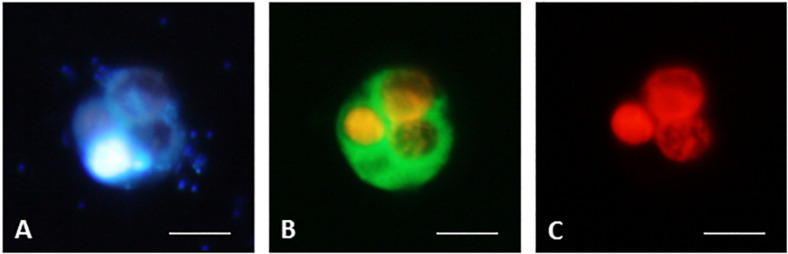
CARD-FISH preparations of ciliate *B. planctonicum* with the probe Bal-651 (Bühler, 2020). Pictures taken with an epifluorescence microscope using appropriate filter setting. **(A)** Cells stained with DAPI; **(B)** Cells at blue light excitation to visualize the green hybridization signal. Orange color comes from autofluorescence of ingested *Cryptomonas* sp.; **(C)** Cells excited with green light to show autofluorescence of ingested *Cryptomonas* sp. Scale bars = 10 μm.

The main strength of the FISH technique is that it is based on SSU rRNA gene phylogeny, and thus, it allows for the direct detection of phylotypes, the presence of which was identified in the samples by sequencing. Probes for novel lineages discovered with the HTS methods can be designed using full-length sequences of the 18S rRNA genes that are identical or have high similarities to short reads obtained via HTS (Piwosz, 2019), obtaining full-length sequence with amplicon-based specific and general eukaryotic primers combined with Sanger sequencing (Gimmler and Stoeck, 2015), or with Nanopore or PacBio long-read sequencing of amplicons (Orr et al., 2018; Davidov et al., 2020; Hatfield et al., 2020). Regions of 18S rRNA unique to the selected, monophyletic group of interest can be then identified in an alignment and a robust phylogeny, at best a phylogenetic inference using the maximum likelihood (ML) criterion (Felsenstein, 1981). Such approach makes it possible to visualize the morphology of novel phylogenetic lineages (Figures 4–6), estimate their abundance and biomass, determine their role in food webs by inspecting food vacuole contents (Figures 4–7), and also reveal symbiotic interaction between different protist species (Figures 4I–L,O), and protists and prokaryotes (Figures 5A–H,P–T, 6A–F,M–Q) (Chambouvet et al., 2008; Dirren et al., 2014; Schulz et al., 2014; Lepère et al., 2016; Piwosz, 2019; Šimek et al., 2020). This powerful approach has completely changed our views on abundance, functions, and dynamics of microbial populations in different environments. We strongly encourage researchers interested in protistan ecology to explore CARD-FISH in combination with other ecological approaches (see examples in Figures 4–7), as they provide taxonomic resolution of trophic interactions between different microbes and address the question “who eats whom” in microbial food webs at a single cell level. Below, we provide a brief discussion of CARD-FISH protocols and an overview of new insights into protistan ecology facilitated by this technique, a detailed description of all steps can be found in Supplementary Files 1–5.

## Card-Fish Basics

In general, fluorescence *in situ* hybridization employs oligonucleotide probes that target short regions (usually 15–25 nucleotides in length) of rRNA genes and bind to a specific sequence of rRNA molecules in intact ribosomes. The first step of the CARD-FISH approach is selecting an already published probe or designing a novel probe for the lineage of interest based on a phylogenetic tree. Although most probes have been so far designed for bacteria and archaea, protists can be targeted as well, requiring only slight modifications in the general probe-design process described in great detail by Hugenholtz et al. (2001) and in Supplementary File 1. Table 1 provides basic information about general probes targeting main protistan lineages, while Supplementary Table 1 gives a comprehensive list of published probes targeting diverse protistan lineages and an evaluation of their specificity and coverage. The probes are labeled with a fluorescent dye, such as CY3 and FITC, or with horseradish peroxidase (HRP) that amplifies the signal from fluorescently labeled tyramides via catalyzed reporter deposition (CARD). The signal from the hybridized probes can be subsequently visualized via epifluorescence microscopy or less frequently by flow cytometry (Amann and Fuchs, 2008). The CARD-FISH protocol itself consists of 10 steps (Figure 3), discussed in detail in Supplementary Files 2–5.

**TABLE 1:** List of oligonucleotide probes developed for protistan lineages, hybridization conditions, and overall recommendation for usage based in their current coverage and specificity (see Supplementary Table 1 for details and additional almost 100 probes for more specific protistan lineages).

Probe name	References	Target group	Sequence (5′-3′)	% form.	Temp. (°C)	Rating
EUK1209R (EUK1195)	Giovannoni et al., 1988	Eukaryota	GGGCATCACAGACCTG	30–40	35	Very good
EUK516	Amann et al., 1990	Eukaryota	ACCAGACTTGCCCTCC	20	35	Very good
EUK309	Sogin and Gunderson, 1987	Eukaryota	TCAGGCTCCCTCTCCGG	0	40	Good
Non-_Bal	Piwosz and Pernthaler, 2010	No Eukaryota	CAAGGTATTAACCCGTGGGATT	25	35	Very good
CON1	Rice et al., 1997a	No Eukaryota	GAGCCTGAGAAACGGC	0	45	Very good
CON2	Rice et al., 1997a	No Eukaryota	GTAATTCCAGCTCCAAT	0	45	Very good
CON3	Rice et al., 1997b	No Eukaryota	CTGCCCTTTGTACACAC	0	45	Very good
KIN 516	Bochdansky and Huang, 2010	Kinetoplastea	ACCAGACTTGTCCTCC	40/55	35	Good
EUK516 Comp.	Bochdansky and Huang, 2010	Competitor for KIN516	ACCAGACTTGCCCTCC	40/55	35	Very good
14F	Edgcomb et al., 2011	Kinetoplastea	CUGCCAGUAGUCAUAUAUGCUUGUUUCAAGGA	20	46	Bad
Rev 14F	Edgcomb et al., 2011	Competitor for 14F	UCCUUGAAACAAGCAUAUAUGACUACUGGCAG	20	46	Bad
Diplo516	Bochdansky et al., 2017	Diplonemea	ACCAGACTTGTCCACC	40	35	Very good
DiploR1792	Morgan-Smith et al., 2013	Diplonemea	GCATTCCTCATTCAAGGA	30	35	Good
PRYM01	Lange et al., 1996	Haptophyta	TCGTAAACGGTCCCTACA	10	46	Bad
PRYM02	Simon et al., 2000	Haptophyta	GGAATACGAGTGCCCCTGAC	40	35	Very good
S-C-DINO-1404	John et al., 2003	Dinoflagellata and related lineages	CCTCAAACTTCCTTGCRTTA	20	55	Bad
SYN-I-1161	Thiele et al., 2014	Syndiniales Group I	TCCTCGCGTTAGACACGC	20	46	Bad
SYN-II-675	Thiele et al., 2014	Syndiniales Group II	CACCTCTGACGCGTTAAT	20	46	Good
Alv01	Chambouvet et al., 2008	Syndiniales Group II	GCCTGCCGTGAACACTCT	40	42	Good
CERC_02	Mangot et al., 2009	Cercozoa	AATACGAGCACCCCCAAC	40	35	Bad
LabY1336	Stokes et al., 2002	Labyrinthulomycetes	AACCCGAAATGTCCCTCTAAGAAG	40	35	Bad
CHLO01	Simon et al., 1995	Chlorophyta	GCTCCACGCCTGGTGGTG	25	46	Bad
NCHLO01	Simon et al., 1995	Competitor for CHLO01	GCTCCACTCCTGGTGGTG	25	46	Bad
CHLO02	Simon et al., 2000	Chlorophyta	CTTCGAGCCCCCAACTTT	40	35	Bad
CRYPT 13	Lepère et al., 2008	Cryptophyceae (nuclear)	CGAAATATAAACGGCCCCAAC	40	35	Bad
Crypto B	Metfies and Medlin, 2007	Cryptophyceae (nuclear) and SA1-3C06	ACGGCCCCAACTGTCCCT	50	46	Good
CHRYSO_01	Mangot et al., 2009	Chrysophyceae	TTTCGGACAAGGAAGACTCG	40	35	Bad

*The overall rating of probes is based on coverage of the target group and number of non-target hits in the guide tree of SILVA database RefNR 138.1 (very good: > 80% coverage, no/almost no non-target hits; good: > 50% coverage, almost no non-target hits; bad: < 50% coverage and/or high number of non-target hits). Taxonomy and nomenclature is based on SILVA classification 138.1. % form – percentage of formamide in the hybridization buffer at the specified temperature (Temp.).*

Sample fixation is the key step for the preservation of protistan cells for the CARD-FISH procedure (Figure 3, step 1). It increases cell permeability, which can be a critical factor for protists with thick and/or complex cellular structures (Ku and Sebé-Pedrós, 2019). Insufficiently fixed cells will degrade and rupture during filtration, while harsh and highly concentrated fixatives can shrink and deform cells (Fried et al., 2002), eventually resulting in the ejection of particles ingested in their food vacuoles (Sherr et al., 1989). More information on different fixatives is available in Supplementary File 2. However, we recommend fixing samples with the neutral Lugol’s solution (final concentration 0.5%) for max. 1 min, followed by an immediate addition of particle-free formalin or PFA (final concentration 1–2%), and complete decolorization with a few drops of 3% sodium thiosulphate solution. Samples preserved this way must not be stored longer than 1 h at room temperature or 24 h at 4°C before filtration.

Samples for CARD-FISH should be filtered with low vacuum underpressure onto white polycarbonate filters that retain microbial cells on their surface (Figure 3, step 2). The filtered sample volume needs to be carefully adjusted to avoid multiple layers of cells, which would hamper microscopic analysis and may cause detachment of cells during embedding in agarose (Figure 3, step 3). The latter step minimizes cell loss during the follow up sample handling steps and incubations in buffers required for the CARD-FISH procedure. So prepared filters can be piled up in a Petri dish and stored at –20°C or at –80°C at least for a few months to years.

In contrast to prokaryotic cells (Pernthaler et al., 2002), enzymatic permeabilization is not required for hybridization of some protists (Bochdansky and Huang, 2010), and it is enough to simply dip the filters into 0.01 M HCl solution, which at the same time inactivates endogenous peroxidase (Figure 3, steps 4–5). We obtained strong signals without additional enzymatic pretreatment for protists with thick cellulose cell walls like chlorophytes (Figure 4A), scales-bearing flagellates such as pedinellids (Figures 4B,C) or those having periplasmic scales such as cryptophytes (Figure 4D). However, obtaining good signals from both the predatory NF and prey (prokaryotes or other protists) in their food vacuoles using double CARD-FISH method depends largely on the permeabilization of the cells so that probes can enter, and this can sometimes be quite tricky (Keeling, 2019; Ku and Sebé-Pedrós, 2019).

Stringent hybridization and washing conditions are of crucial importance for obtaining the specific binding of probes. They need to be experimentally optimized for each probe. Stringency of the hybridization at a certain temperature is determined by the concentration of formamide in the hybridization buffer and an appropriate concentration of NaCl in the washing buffer (Pernthaler et al., 2004). The standard duration of the hybridization step (Figure 3, step 6) is 3 h (Not et al., 2002; Piwosz and Pernthaler, 2010), but its prolongation (even up to 4 days) may improve the signal intensity from less active cells (Lim et al., 1993; Amann and Fuchs, 2008; Morgan-Smith et al., 2013; Mukherjee et al., 2015). The washing step (Figure 3, step 7) is done at temperature 2°C higher than the hybridization. It removes probes weakly hybridized to non-target cells and also non-hybridized probes, so they do not produce false positive signals during the CARD step.

Catalyzed reporter deposition or tyramide signal amplification (TSA; Figure 3, step 8) enhances the fluorescent signals by deposition of a large number of fluorescently labeled tyramines (Bobrow et al., 1991). There is a wide array of available dyes, but those emitting green light (emission maximum at 520 nm), such as Fluorescein isothiocyanate (FITC) or Alexa488, are the most commonly used (see Figures 4–6). In double CARD-FISH, two probes targeting two different microbial populations are used subsequently in the same filter section on the same microscopic slide. The choice of fluorescent colors is quite critical for visualization and differentiation of targeted cells with this approach. We advise, based on our experience, to use brighter green or yellow colors for detection of prey in the food vacuoles, and darker blue, orange, or red colors for HF or blue or far red for MF that show chlorophyll autofluorescence in orange and red. The available dyes are discussed in Supplementary File 2. Examples of the well distinguishable color combinations are shown in Figures 6A–H.

Hybridized and dried filter pieces need to be counterstained with a DNA dye, such as commonly used DAPI stain (Coleman, 1980; Porter and Feig, 1980), which can be added to a glycerol containing anti-fading mixture like Vectashield (Vector laboratories), used to mount the samples on microscopic slides and to reduce the signal fading (Figure 3, step 9). Hybridized protistan cells are counted as percent of all protists detected with a general nucleic acid stain. Independent total NF counts are required to estimate absolute numbers of the targeted HF and MF lineages. However, the absolute number of protists should be assessed from counts obtained with general eukaryotic probes, because nuclei of small protists can be mistaken for prokaryote cells (Beardsley et al., 2005; Pernice et al., 2014). Moreover, the contribution of specific lineages to higher taxonomic levels can be estimated as well. Counting precision (expressed as 95% confidence limits) depends on the number of counted hybridized cells and the number of counted fields of view. It is ±20% for 100 counted cells, 10% for 400 cells, and 6% for thousand cells (Edler and Elbrächter, 2010). However, such numbers may be impossible to reach for rare taxa, and thus, we recommend counting 500–1000 cells counterstained with a DNA dye. Alternatively, percent proportion of counted cells hybridized with a specific probe can be plotted against the number of counted DNA-stained cells until the plateau is reached, indicating the sufficient counting effort. Flow cytometry or automatic image acquisition microscopy may allow for statistically robust abundance estimates of rare lineages (Simon et al., 1995; Biegala et al., 2003; Mangot et al., 2018).

Unsuccessful CARD-FISH procedure can be identified by weak or lack of signals, high background fluorescence, and unspecific probe binding that can be recognized by diverse shape and size variability of hybridized cells, especially in case of very specific probes. For detailed troubleshooting guide, please refer to the Supplementary File 5.

### Limitations of the Method

As we show below, CARD-FISH is a powerful tool to unveil ecological traits of poorly studied and uncultured protists in their natural environments at a single cell level. However, as every method, it has its limitations. First, sample fixation might results in a certain cell loss, and fixatives do not have uniform efficiency for all protists (Jeuck et al., 2017). Moreover, CARD-FISH accuracy may be compromised by imperfect probe coverage and specificity, differences in the amount and activity of endogenous peroxidases between phylogenetic groups and environmental samples, and poor detection of low abundance or inactive community members, and difficulties in counting aggregated cells (Pernthaler et al., 2004; Amann and Fuchs, 2008). Moreover, tyramides sometimes bind unspecifically to cell walls of some protists, such as dinoflagellates or diatoms, making microscopic evaluation more challenging. Autofluorescence of chloroplasts might also interfere with probe signals depending on used fluorochromes and filter sets of the epifluorescence microscope used for quantification (Figure 7, Bühler, 2020). However, appropriate fluorochrome combinations considerably improve distinction of such objects as exemplified in Figures 4B,D, 5K,L. Other parameters may reduce the fluorescence of target cells, in particular a low rRNA content linked to low growth rate (Lim et al., 1993; Amann et al., 1995; Simon et al., 1995; Head et al., 1998). Nevertheless, most of these issues can be addressed as described in Supplementary Files 1, 5.

## New Insights Into Morphology, Physiology, and Ecology of Uncultured and Little Known Protists From Card-Fish and Double Card-Fish Applications

Understanding the *in situ* distribution and seasonal dynamics of individual protistan groups is important for obtaining a clear picture of the microbial processes in any ecosystem (Sherr et al., 2007; Kim et al., 2014). Although analyses of samples by CARD-FISH and double CARD-FISH is laborious, these single-cell approaches have revealed exciting discoveries on the importance of various flagellate taxa in marine and freshwater pelagic food webs. They have provided completely new insights into the life strategies of so far unknown or morphologically indistinguishable protists and will help to elucidate yet unknown trophic interactions of uncultured protists (Figures 4–6) that form highly complex microbial food webs. Below, we shortly summarize some key discoveries enabled by these techniques.

### Novel HF and MF in Marine Environments

One of the first surprises upon applying FISH to marine samples was that the described species of bacterivorous HF were not abundant in natural environments. For instance, the genus *Paraphysomonas* was found to contribute below 1% to the total abundance of HF (Lim et al., 1999). In contrast, novel lineages of marine Stramenopiles (MAST), especially nanoplanktonic MAST-1 (cell size: 4–7 μm) and picoplanktonic MAST-4 (cell size: 2–3 μm), turned out to be ubiquitous in open oceans, contributing together to about 20% of all HF (Massana et al., 2006b; Mangot et al., 2018), and exhibiting growth rates between 0.4 and 0.8 per day (Massana et al., 2006a). Two morphotypes of the MAST-6 lineage were observed in the brackish Baltic Sea: larger (approximately 15 μm, Figure 4E) that formed a conspicuous peak when the salinity dropped to 6.2, and smaller (around 6 μm, Figure 6F) that dominated at salinities > 7 (Piwosz and Pernthaler, 2010). The MAST-2 lineage (cell size 4–5 μm) was found to be more abundant in first-year sea-ice (2–10%, Piwosz et al., 2013) than in pelagial (0.3%, Massana et al., 2006b). In contrast, cercozoans of the genus *Cryothecomonas* (cell size 2.5–29 μm) were also found in the sea-ice, but in rather low abundances (Thaler and Lovejoy, 2012). Another lineages of Cercozoa from Novel Clade 2 (Bass and Cavalier-Smith, 2004) turned out to be rare in the brackish Baltic Sea (Figures 6G–L), but they exhibited rapid growth rates of >1 per day in experimental incubations (Piwosz and Pernthaler, 2011).

Another unexpected discovery was that parasitic HF may appear in high concentrations in pelagic environments. Members of the order Syndiniales have been shown to control blooms of toxic dinoflagellates that form red-tides (Chambouvet et al., 2008, 2011). Free-living dinospores or multicellular trophonts were also found in the Baltic Sea (Piwosz and Pernthaler, 2010), where they may infect a bloom forming dinoflagellate *Heterocapsa triquetra* (Figures 4G,H), and in the first-year sea-ice (Piwosz et al., 2013). Syndiniales are more abundant in coastal waters, where they can contribute up to 40% of all eukaryotes and infect up to 25% of the target dinoflagellate species (Siano et al., 2011). In contrast, parasitic fungi were shown to be important nanophytoplankton parasites in the open ocean, where they can contribute up to 14% of abundance of all eukaryotes and infect up to 12% of haptophyte and 6% of chrysophyte algae (Lepère et al., 2016).

Completely different eukaryotic communities were found using CARD-FISH in the deep sea below 1000 m depth, where seven groups contributed to 50–70% of all eukaryotes: kinetoplastids (7–20%), labyrinthulomycetes (2–25%), fungi (2–25%), diplonemids (1–3%), Syndiniales group II (2–8%), MAST-4 lineage (1–6%), and an unidentified HF with a peculiar nuclear morphology (Edgcomb et al., 2011; Morgan-Smith et al., 2011, 2013). Fungi and labyrinthulomycetes seem to be more abundant on marine snow particles (Bochdansky et al., 2017) and in oxygen minimum zones (Morgan-Smith et al., 2013). In contrast to HF from the sun lit ocean, deep sea HF, especially those living on marine snow, might be saprotrophic rather than bacterivorous (Bochdansky et al., 2017).

The signal amplification was of crucial importance for detection of algae and MF showing strong autofluorescence from chlorophylls that may mask weak signals of monolabeled probes (Medlin and Strieben, 2010) (Figures 4A,B,D). Chlorophytes (Figure 4A), predominantly genus *Micromonas*, turned out to dominate the abundance of picophytoplankton in coastal oceans and seas, with contributions exceeding 80% (Not et al., 2005, 2008; Thiele et al., 2014; Unrein et al., 2014; Piwosz et al., 2015; Cabello et al., 2016; Piwosz, 2019). Their contribution was below 40% in other marine habitats, such as surface waters or deep chlorophyll maxima in the open oceans, Arctic fjords or the first-year sea-ice (Not et al., 2008; Piwosz et al., 2013, 2015). Larger haptophytes (cell sizes between 2–6 μm, Cabello et al., 2016; Piwosz, 2019) are the second most abundant group in the marine waters, with contributions to nanophytoplankton abundance of about 15% in the open sea, up to 30% in the brackish Baltic Sea, and above 35% in the polar regions (Not et al., 2005, 2008; Piwosz, 2019). Genus *Pheocystis* and family Pavlovales substantially contribute to haptophyte numbers in the open ocean (Thiele et al., 2014; Piwosz et al., 2015) and sea ice (Piwosz et al., 2013), while genera of *Haptolina* and *Chrysochromulina* were abundant in the brackish Baltic Sea (Piwosz, 2019). Pelagophyceae were found to be typically cells < 3 μm and contributing more to the deep chlorophyll maximum (about 24%) than in surface waters (below 10%, Cabello et al., 2016), but were of little importance in the brackish waters (Piwosz, 2019). In contrast, cryptophytes (Figure 4D) seem to be more important in coastal brackish waters (Piwosz et al., 2016; Piwosz, 2019) or in sea-ice (Piwosz et al., 2013) than in open ocean (Piwosz et al., 2015). A set of probes targeting photosynthetic cryptophyte clades has been developed and optimized for CARD-FISH (Table 1 and Supplementary Table 1, Metfies and Medlin, 2007; Medlin and Schmidt, 2010). However, the most abundant cryptophytes are members of the heterotrophic CRY1 lineage (Figures 5A–D), at least in the coastal waters of the Baltic Sea and freshwater habitats (Piwosz et al., 2016; Shiratori and Ishida, 2016; Grujčić et al., 2018; Piwosz, 2019; Šimek et al., 2020). Finally, bolidophytes and pedinellids seem to be rare (<1%) in truly marine waters (Guillou et al., 1999; Beardsley et al., 2005; Not et al., 2005; Piwosz et al., 2015), but the latter group sometimes form blooms in the Baltic Sea (Figures 4B,C, Piwosz and Pernthaler, 2010; Piwosz, 2019).

### Novel HF and MF in Freshwater Lakes

Freshwater lakes are much more accessible than open oceans, thus they are ideal ecosystems to understand the seasonal dynamics of small protists (Simon et al., 2015a; Mukherjee et al., 2017). However, *in situ* distributions, including vertical water column abundance patterns, and seasonal dynamics of protists remain still poorly studied in freshwater lakes, and only a handful of lineages have been quantified using CARD-FISH probes (Mangot et al., 2009; Mukherjee et al., 2017; Grujčić et al., 2018; Mukherjee et al., 2019; Šimek et al., 2020). Nevertheless, similar to marine environments, even a small number of such studies have brought unexpected results. One of the biggest surprises was the finding that the most abundant and omnipresent HF in various freshwater and even brackish habitats are not so called “*Spumella*-like” chrysomonads (Šimek et al., 1997a; Boenigk and Arndt, 2002; Matz et al., 2002; Grossmann et al., 2016), but tiny aplastidic cryptophytes and especially the cryptophyte CRY1 lineage therein (Piwosz et al., 2016; Shiratori and Ishida, 2016; Grujčić et al., 2018; Šimek et al., 2020). Concerning their typical cell size between 3 and 6 μm (Figures 5A–D), CRY1 were initially suggested to be bacterivores (Piwosz et al., 2016), which was later confirmed with observations of visibly ingested bacteria (Figures 5A–D, 6A–C) in their food vacuoles (Grujčić et al., 2018; Šimek et al., 2020). In different seasonal aspects of a freshwater reservoir, the aplastidic cryptophytes and cryptophyte CRY1 lineage accounted for, on average, ca. 50% and 20–25% of total HF numbers, respectively (Grujčić et al., 2018; Šimek et al., 2020). Moreover, the application of FLBs in combination with CARD-FISH clearly demonstrated their high bacterial consumption rates (Figures 6M–Q). In contrast, plastidic cryptophytes (Figure 4D) are important members of spring phytoplankton blooms, where they can account for up to 15% of all eukaryotes (Mangot et al., 2009), but they mostly do not show any uptake of prokaryotes.

Kinetoplastids are another surprisingly common and abundant lineage of HF in freshwaters. These flagellates are known as poor swimmers, being associated with detritus (Caron et al., 1982; Zubkov and Sleigh, 2000), where they glide around the particles to feed on surface-associated bacteria (Boenigk and Arndt, 2000). They were known to be ubiquitous in aquatic ecosystems (von der Heyden and Cavalier-Smith, 2005; Simpson et al., 2006), and they were even detected using light microscopy (Brandt and Sleigh, 2000; Weitere and Arndt, 2003). However, only upon application of CARD-FISH and kinetoplastid-specific probes (Table 1 and Supplementary Table 1), these flagellates were found to be widely distributed in freshwater lakes and, similarly to their deep ocean counterparts, to dominate in deep oxygenated hypolimnion waters, especially in summer when their contribution to total abundance of protists can reach up to 54% (Mukherjee et al., 2015, 2019). The timing of kinetoplastids maxima in the hypolimnetic waters was linked to the termination of phytoplankton blooms and high numbers of detritus particles sinking from the surface waters. The appearance of kinetoplastids in the surface waters may also correspond to the end of phytoplankton blooms, but it seems to be low in all the seasons (Mukherjee et al., 2015). However, their seasonal contributions to total HF in hypertrophic ponds, rich in small algae and detrital particles, were rather high, with examples of their typical bacterivorous morphotypes with well visible nuclei, kinetoplasts, and food vacuole contents shown in Figures 5Q–T.

Interestingly, a sister group of kinetoplastids, diplonemids, have been considered an exclusively marine group until a recent discovery of a distinct freshwater lineage widespread in various freshwater lakes around the world (Mukherjee et al., 2019, 2020) (Figures 4J–L). Their diversity seems to be low compared to the marine lineages and due to their low abundance, a clear seasonal pattern could not be deduced using CARD-FISH probes (Mukherjee et al., 2020). However, all the probe-positive cells clearly showed several bacteria inside their cytoplasm indicating their bacterial uptake (Mukherjee et al., 2020).

Cercozoans are smaller heterotrophic protists (size range 2–20 μm) belonging to Rhizaria (Figure 1A). Large Rhizaria are among the most dominant group of protists in the world oceans (Biard et al., 2016), but the abundance and distribution patterns of small cercozoans are less understood (Bass and Cavalier-Smith, 2004). Application of CARD-FISH revealed that cercozoans are consistently present in freshwater lakes, contributing around 11–12% of total protists (Mangot et al., 2009; Lepère et al., 2010), and they can ingest even cyanobacterial *Microcystis* cells (Figure 5K). Cercozoans of Novel Clade 7 (Bass and Cavalier-Smith, 2004) can account for up to 28% of the total community in food-web manipulation experiments amended with bacterial prey, which induced rapid growth of small HF and in turn also the growth of the predatory cercozoans with well visible small prey protists in their food vacuoles (Šimek et al., 2020) (Figures 5I,J).

Chlorophytes, cryptophytes, and haptophytes have also been reported to be consistently present throughout the year in freshwater lakes, mainly in the epilimnion waters, where their contribution to total eukaryotes can be > 50% (Mangot et al., 2009; Lepère et al., 2010). Autotrophic chlorophytes and haptophytes are considered as typical components of summer phytoplankton blooms in various lakes (Sommer et al., 1986, 2012). Chrysophytes are another dominant group of freshwater protists (del Campo and Massana, 2011) and are consistently present year round in freshwater lakes contributing up to 35% of total CARD-FISH positive eukaryotes (Mangot et al., 2009).

Parasitic perkinsozoans are regularly reported from freshwater lakes (Lepère et al., 2008, 2010; Mangot et al., 2011; Mukherjee et al., 2017). Their abundance peaks during summer, where they can account for up to 31% of the total eukaryotic community (Mangot et al., 2009). Perkinsea have been recently shown to infect green algae of the genus *Sphaerocystis*, and cells attached to filamentous cyanobacteria were also observed (Jobard et al., 2019). These parasites made up to 24% of all protists in the hypolimnion of a reservoir, where they were found attached to unidentified protistan cells and lake snow (Figure 4I).

### Grazing Preferences and Grazing Rates of Protistan Lineages

Newly designed probes, targeting *in situ* morphologically indistinguishable HF and MF, have brought invaluable new information on absolute numbers of particular protistan lineages with the possibility to inspect also their food vacuole contents, thus unveiling their feeding modes (see examples in Figures 4–7) and examining grazing and growth rates of specific bacterivorous HF and MF. The method to estimate bacterivory based on FLB uptake rate during short-term incubations (Sherr et al., 1987) can be combined with identification of the grazers via CARD-FISH using the protocol described in Supplementary Files 2–4. This approach has unveiled that heterotrophic members of Cryptophyceae and its CRY1 lineage are important freshwater pelagic bacterivores (Grujčić et al., 2018; Šimek et al., 2020) (Figures 5A–H, 6A–F,M–Q). In marine waters, FISH combined with FLBs enabled detecting that HF of the MAST-1C lineage have higher grazing rates (∼4 bacteria HF^–1^ h^–1^) than members of the MAST-4 lineage (<1.5 bacteria HF^–1^ h^–1^), with an overall average cell-specific rate slightly above 2 bacteria HF^–1^ h^–1^ (Massana et al., 2009). Marine haptophytes were also found to be important bacterivores, with ingestion rates > 2.8 bacteria MF^–1^ h^–1^ and contributing even more than 25% to total protistan bacterivory (Unrein et al., 2014).

Furthermore, CARD-FISH enabled to reveal how bacterial food characteristics modulate growth and community dynamics of major bacterivores and consequently carbon flow rates to higher trophic levels. Monitoring abundance of specific HF lineages using CARD-FISH has evidenced that their growth response differs depending on a bacterioplankton species available as a main food source (Grujčić et al., 2018; Šimek et al., 2018, 2020). For instance, compositional shifts in bacterivorous HF communities tracked by CARD-FISH (Grujčić et al., 2018) cascaded through the food chain even to the level of predatory and omnivorous HF and small ciliates (Šimek et al., 2020).

The double CARD-FISH technique provides even higher resolution by allowing simultaneous phylogenetic identification of both predator and prey (Massana et al., 2009; Piwosz and Pernthaler, 2011; Grujčić et al., 2018; Šimek et al., 2020), thus ultimately opening the “black box” of microbial food webs and addressing the intriguing questions “who eats whom” (see examples in Figure 6). Ingested bacteria can be identified directly in food vacuoles (Figures 5, 6), providing information on positive or negative selections for particular bacterial and archaeal phylotypes (Jezbera et al., 2005, 2006; Massana et al., 2009; Anderson et al., 2012; Gerea et al., 2013; Šimek et al., 2014, 2020; Ballen-Segura et al., 2017).

Finally, newly designed CARD-FISH probes also shed light on the feeding modes of medium sized (5–20 μm) HF. Trophic relationships between bacterivorous cercozoans of the Novel Clade 2 (Bass and Cavalier-Smith, 2004) targeted by probe Cerc_Bal01 (Figure 6L), larger, omnivorous predator also of the Novel Clade 2 targeted by probe Cerc_Bal02 (Figure 6G), and heterotrophic dinoflagellates (Figure 6I) were deduced from experimental manipulations of a natural plankton community from coastal waters of the brackish Baltic Sea (Piwosz and Pernthaler, 2011). Similarly, experiments with a freshwater plankton community led to enrichments of 7–12 μm large HF affiliated with cercozoan Novel Clade 7 (Figures 5I,J) (Bass and Cavalier-Smith, 2004) and uncultured kathablepharids (Figures 5M–P) that grazed on small aplastidic bacterivorous cryptophytes or small algae rather than bacteria (Šimek et al., 2020). Time-course data of different HF lineages also allow for calculating lineage-specific growth rates, typically ranging from 1 to 1.8 d^–1^ for bacterivorous cryptophytes (Figures 5E–H), their CRY1 lineage (Figures 5A–D), and kinetoplastids (Figures 5Q–T), but also for larger omnivorous and predatory kathablepharids (Figures 5M–P) and Cercozoa (Figures 5I,J) (Šimek et al., 2020). Similarly, consumption rates on bacteria or probe-targeted lineages of prey protists can be estimated (Šimek et al., 2020). For instance, a comparison of cell biovolumes of prey cells from the CRY1 lineage (Figures 5A–D) with their cercozoan predator from the Novel Clade 7 (Figures 5I,J), and also taking into account the growth rate of the predatory cercozoans (doubling time of 10–20 h) indicated that these predators had to ingest ∼25–45 small CRY1 flagellates to meet their carbon requirements per one doubling (Šimek et al., 2020). Moreover, the rapid growth of both bacterivorous heterotrophic cryptophytes and omnivorous cercozoans and kathablepharids (in hours to days) tightly corresponded to typical doubling times reported for fast growing bacterioplankton groups (Eckert et al., 2012; Šimek et al., 2014; Neuenschwander et al., 2015). These findings allowed proposing a conceptual model explaining the tight linkages between rapid bacterial community shifts and succeeding HF community shifts in freshwaters (Šimek et al., 2018), now detectable using specific FISH probes (Figures 4–6).

## A New Model for Aquatic Microbial Food Webs

**FIGURE 8:**
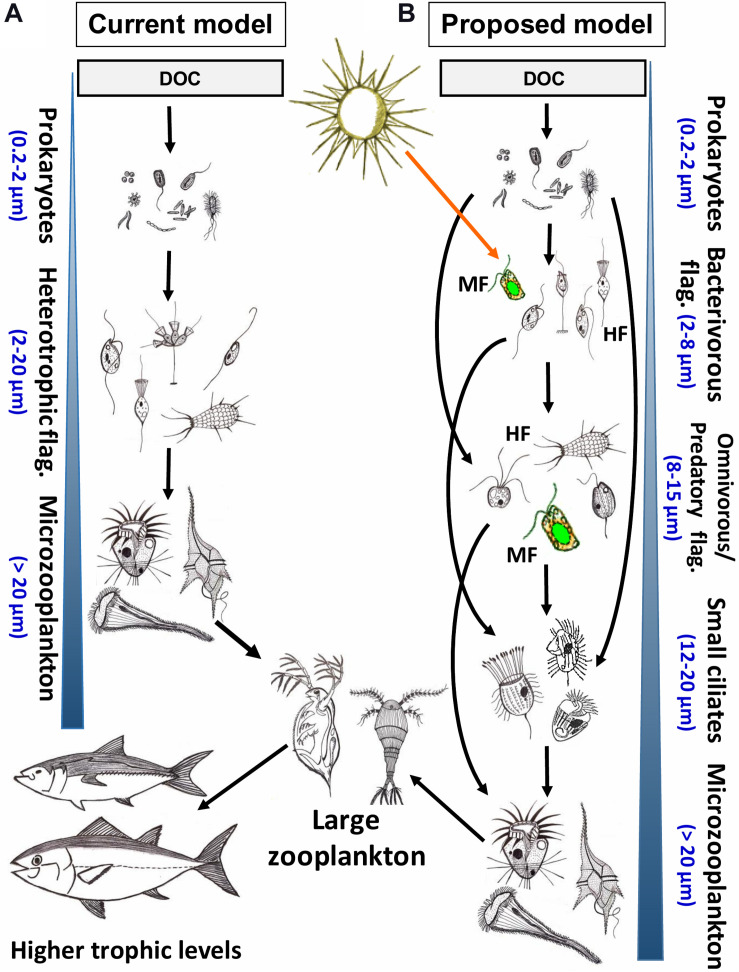
Conceptual models of aquatic microbial food webs aimed at describing major feeding and predatory relationships in pelagic systems. For simplicity of depiction, viruses, parasites and primary producers (phytoplankton) are not included, their roles have been reviewed recently elsewhere (Suttle, 2007; Winter et al., 2010; Sommer et al., 2012; Grossart et al., 2019; Zimmerman et al., 2020). **(A)** The original, most commonly applied model of major trophic interactions in microbial communities assumes generally linear carbon fluxes between neighboring trophic levels in the trophic food chain (Azam and Malfatti, 2007). All nanoplanktonic flagellates (NF) are considered to be primarily heterotrophic bacterivores that are grazed by microplanktonic protists, mainly ciliates and dinoflagellates. **(B)** Our proposed updated model of microbial communities includes recently recognized importance of novel trophic relationships within the nanoplankton size fraction (2–20 μm) described and also extensively documented in this review (see Figures 4–6). In this model, prokaryotes are grazed generally by small (2–8 μm) HF and MF. Larger NF (8–15 μm) are either omnivores (grazing on both bacteria and small NF) or predators (grazing mostly on small NF). However, these omnivorous and predatory NF prey also on each other (indicated by a blue circular arrow), and are themselves grazed by small filter-feeding and predatory ciliates (12–20 μm), which might also consume bacteria and bacterivorous NF. The larger omnivorous and predatory NF and small ciliates are controlled mainly by microplankton size fraction dominated by ciliates and dinoflagellates. Microzooplankton is consumed by mesozooplankton, thus channeling the carbon flow to higher trophic levels. Blue arrowhead indicated increasing cell- or body-size of planktonic organisms within a microbial community at different trophic levels. DOC, dissolved organic carbon. HF and MF, heterotrophic and mixotrophic flagellates, respectively.

The current view of aquatic microbial food webs assumes a relatively simple transfer of energy from prokaryotes via HF and MF to ciliates, and finally to zooplankton (Figure 8A). HF and MF are typically considered to prey predominantly on prokaryotes, even though it has been repeatedly shown that major bacterivores are actually those smaller than 5–8 μm (e.g., Chrzanowski and Šimek, 1990; Šimek et al., 1997b; Jürgens and Matz, 2002). The discovery of an enormous diversity in taxonomy, morphology, feeding modes, and life strategies of nanoflagellates provides compelling evidence that microbial food webs are far more complex than initially proposed, and the classical model requires a considerable revision in the light of recent findings (Arndt et al., 2000; Massana et al., 2009; Piwosz and Pernthaler, 2011; Grujčić et al., 2018; Šimek et al., 2020). The major obstacles in assigning the appropriate trophic roles to middle-sized flagellates in planktonic habitats are: (i) limited morphological features, which hamper rapid and reliable taxonomic identification by microscopy (Jeuck and Arndt, 2013), and (ii) a limited possibility to visualize and characterize ingested prey in food vacuoles of the protistan cells in natural communities that would indicate their food preference (Jezbera et al., 2005; Šimek et al., 2020). CARD-FISH based approaches are a perfect tool to solve these hindrances at a single cell level, as we showed above and exemplified in numerous microphotographs (Figures 4–7). Middle-sized NF (8–15 μm) are either omnivores feeding simultaneously on bacteria, algae, and bacterivorous HF and MF, or specific predators feeding primarily on motile algae and bacterivorous HF and MF (Piwosz and Pernthaler, 2010, 2011; Šimek et al., 2020). In general, Cercozoa and Katablepharida seem to be among the key consumers of bacterivorous HF and MF, at least in freshwaters and coastal brackish waters (Piwosz and Pernthaler, 2011; Grujčić et al., 2015; Mukherjee et al., 2017, 2019; Šimek et al., 2020). A single predatory cercozoan cell from Novel Clade 7 with an estimated doubling time of ∼12–24 h consumes daily 35–50 bacterivorous HF (Šimek et al., 2020). Similar doubling times were observed for predatory cercozoans from Novel Clade 2 (Piwosz and Pernthaler, 2011). These growth rates point to the dynamics and crucial role of the proposed trophic links to the overall energy and carbon transfer rates in microbial food webs. Similarly, small ciliates (12–20 μm), currently assumed to be primarily omnivorous, actually belong to the key planktonic bacterivores in most eutrophic and hypertrophic freshwater habitats (Šimek et al., 2000, 2019; Posch et al., 2015). These trophic interactions significantly modulate carbon flow efficiency, but their contribution to energy transfer in aquatic ecosystems is neither understood nor well quantified (Diehl and Feiáe, 2000).

To provoke a further debate on this topic and to advance our understanding of these interactions, we propose a new, considerably more complex but also more realistic model of microbial food webs (Figure 8B). The major refinement is related to the fact that the seemingly single level link (generally attributed to bacterivorous MF and HF) between prokaryotes and microzooplankton (mainly ciliates and dinoflagellates) actually consists of multiple linear and parallel trophic levels of omnivorous and predatory protists in the size range of ca. 8–15 μm. Recognition of these additional trophic levels and their complexity is the first step to quantify the contribution of phylogenetically and functionally diverse HF and MF in pelagic food webs. Consequences of additional trophic levels generally imply that more carbon is dissipated as CO_2_ within the microbial food web, and thus, a less efficient carbon transfer to higher trophic levels is anticipated. On the other hand, the energetic consequences of omnivory follow the opposite direction, as it reduces the number of trophic steps, resulting in more direct carbon flow from microbes to higher trophic levels (Goldman and Caron, 1985; Šimek et al., 2020). Omnivory has been also suggested to stabilize dynamics of food webs and communities (Fagan, 1997; McCann and Hastings, 1997; Holyoak and Sachdev, 1998).

## Outlook: Future Developments and Applications of Card-Fish to Protists

CARD-FISH opens the door to a new era for studying small MF and HF by revealing their identity and feeding modes, thus providing novel insights into the ecology of otherwise poorly distinguishable groups. Moreover, it may also advance our understanding of the ecology of groups with generally larger cell sizes, such as ciliates or dinoflagellates. These groups are morphologically highly diverse, and a battery of methods has been developed to visualize characteristic features using light microscopy and numerous taxonomic keys, but reliable identification of smaller species requires substantial experience and it is generally time-consuming (Montagnes and Lynn, 1987; Foissner and Berger, 1996; Foissner et al., 1999; Madoni, 2011). So far, FISH-based identification has been proposed for economically important species and genera, such as the toxic dinoflagellates *Alexandrium* and *Azadinium* (John et al., 2003; Touzet et al., 2010; Toebe et al., 2012), or the pathogenic ciliate *Pseudocohnilembus persalinus* (Zhan et al., 2014), but it has not been routinely applied in ecological studies yet. FISH with monolabeled probes was successfully combined with Protargol staining and silver nitrate impregnation methods (Fried et al., 2002). Recently, a promising FISH and CARD-FISH protocol for freshwater planktonic ciliates has been developed (Bühler, 2020). This protocol was reliable for cultured ciliate strains and in multispecies mock assemblages (Figure 7, courtesy of D. Bühler and T. Posch). However, the FISH signal to noise ratio was insufficient for reliable *in situ* quantification. Even so, CARD-FISH proved to be a promising tool for the *in situ* detection and enumeration of tiny, barely distinguishable planktonic ciliates (Bühler, 2020).

CARD-FISH can be also used to study ecology of cryptic species. Multiple ribosomal lineages within one morpho-species have been discovered in many groups, such as dinoflagellates, diatoms, ciliates, and diplonemids (Zhu et al., 2005; Potvin and Lovejoy, 2009; Santoferrara et al., 2014; Stoeck et al., 2014; Caron and Hu, 2019; Mukherjee et al., 2020). Cryptic species differ in their eco-physiological characteristics, for instance, toxin production, as observed for dinoflagellate *Alexandrium tamarense* in the North Sea (Töbe et al., 2013), or habitat preference of *Micromonas pusilla* (Šlapeta et al., 2006). Such studies would enhance not only our understanding of protistan ecology and functional role in an ecosystem but also of their evolution and adaptation to different environments.

Fluorescence *in situ* hybridization-based methods are constantly being modified and developed. Recently, simultaneous analysis of even hundreds of lineages has been made possible with multi-color FISH approaches combined with high resolution confocal microscopy (Valm et al., 2011; Lukumbuzya et al., 2019; Shi et al., 2020). These novel techniques could be used, for example, to analyze food vacuole content for the presence of different prey items in multiple protistan lineages simultaneously, allowing for more direct determination of food preferences, and likely also accelerating sample processing. Moreover, FISH methods such as GeneFISH that combines the detection of specific genes and ribosomal RNA (rRNA) at the single cell level (Moraru et al., 2010) and mRNA FISH that detects synthesis and stability of mRNAs (Wendeberg et al., 2012; Xie et al., 2018) have great potential to be applied on protists. For example, with mRNA FISH, it may be possible to assess how localization patterns of mRNAs change during different stages of protist feeding on bacteria.

CARD-FISH can be also applied to samples gained from soils and sediments, where elevated organic matter content may cause high background, as shown for prokaryotes (Ferrari et al., 2006; Eickhorst and Tippkötter, 2008). As in the case of nanoplanktonic MF and HF, CARD-FISH protocols likely can be optimized for soil protists. Due to a lack of experience, we cannot recommend specific solutions, but considering the recent discovery of the impact of protistan grazing on decomposition rate (Geisen et al., 2020) or putatively high abundances of parasitic apicomplexans in soils (Mahé et al., 2017), the application of CARD-FISH might open a new universe of protistan ecology in soils and sediments, too.

Finally, we also suggest using various experimental manipulations with natural microbial communities (in order to considerably enrich the target protistan populations) in combination with novel probe design for both eukaryotic grazers and prokaryotic prey as highly relevant and useful approaches (Massana et al., 2009; Piwosz and Pernthaler, 2011; Šimek et al., 2013, 2020; Grujčić et al., 2018). This methods’ combination provides completely new insights into the life strategies of so far unknown or morphologically indistinguishable protists and to elucidate yet unknown trophic interactions and feeding modes (see examples in Figures 4–6) of uncultured nano-sized protists that form highly complex microbial food webs (Figure 8B). The current level of understanding of microbial interactions would undoubtedly profit from more frequent applications of FISH and other single cell approaches to considerably deepen our so far only mosaic knowledge on the ecology of most aquatic protists.

## Author Contributions

KP and KŠ conceptualized the study. All authors contributed to drafting the manuscript, preparing the figures, making critical revisions, and approved the final version of the manuscript.

## Conflict of Interest

The authors declare that the research was conducted in the absence of any commercial or financial relationships that could be construed as a potential conflict of interest.

## Supplementary Material

Supplementary Figure 1 Ready-to-print examples of circles for cutting filters into sections.

Supplementary File 1 Guide for oligonucleotide probe design for protists.

Supplementary File 2 Detailed description of the recommended CARD-FISH protocol.

Supplementary File 3 Single page versions of the recommended CARD-FISH and double CARD-FISH protocols.

Supplementary File 4 Recipes for all buffer, solutions and reagents used during CARD-FISH procedure.

Supplementary File 5 Troubleshooting guide.

Supplementary File 6 Reference list for Supplementary Table 1.

Supplementary Table 1 List of FISH and CARD-FISH probes developed for protistan lineages, hybridization conditions, their current coverage and specificity, and overall recommendation for usage. The overall rating of probes is based on coverage of the target group and number of non-target hits in the guide tree of SILVA database RefNR 138.1 (very good: >80 coverage, no/almost no non-target hits; good: >50% coverage, almost no non-target hits; bad: <50 coverage and/or high number of non-target hits). Taxonomy is based on SILVA classification 138.1. List of references is available as Supplementary File 6. If two references to one probe are given, the first corresponds to the work that developed the probe, and the second to the work that tested it for FISH/CARD-FISH application.

Supplementary Table 2 Volumes of formamide (form.) and water for 20 ml of hybridization buffer (HB), volumes of 5 M NaCl in 50 ml of washing buffer at 37°C (for hybridization at 35°C) and 48°C (for hybridization at 46°C). The recipes for HB and WB can be found in Supplementary File 4.

Supplementary Table 3 Composition of buffers according to two most commonly used protocols: Not et al. (2002) and Piwosz and Pernthaler (2010). Concentrations of formamide in the hybridization buffer need to be optimized for each probe. The detailed instructions on how to prepare the buffers are given in Supplementary File 4.
