# Supplementary material for: CARD-FISH in the Sequencing Era: Opening a New Universe of Protistan Ecology
Source: Front Microbiol. 2021 Mar 4;12:640066. doi: 10.3389/fmicb.2021.640066 (PMC7970053; doi:10.3389/fmicb.2021.640066)
Supplement: Supplementary Figure 1 — Ready-to-print examples of circles for cutting filters into sections. [file Image_1.PDF]

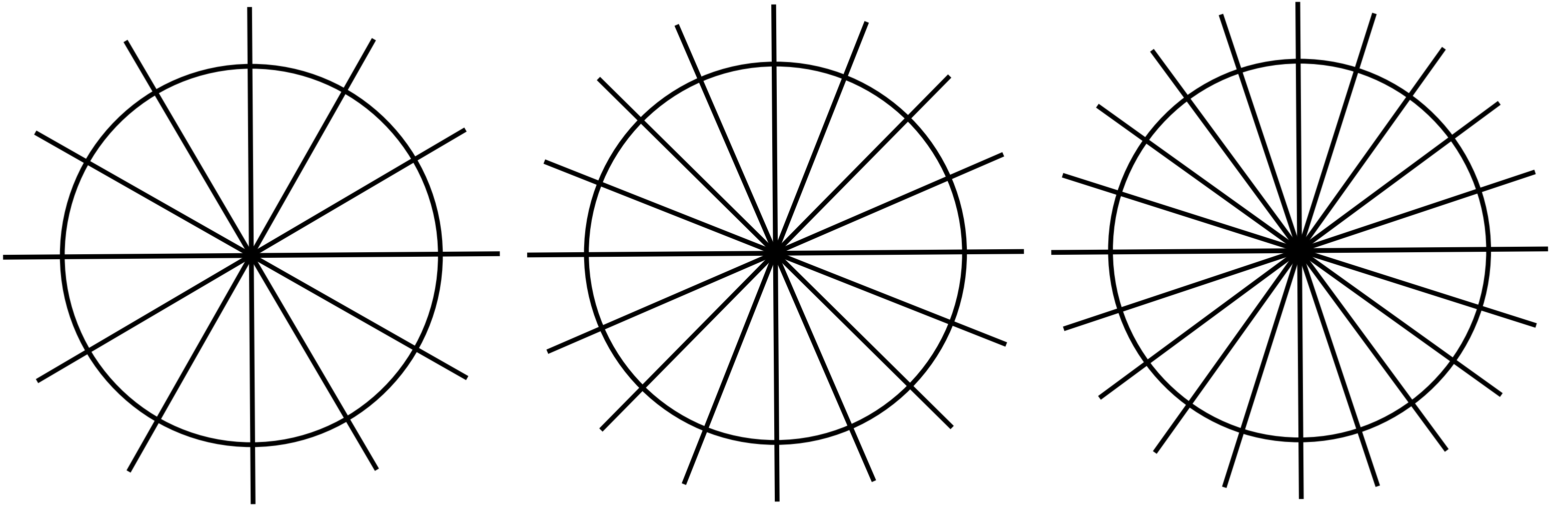

12 sections

16 sections

20 sections

Supplementary Figure S1: Ready-to-print examples of circles for cutting the filters into sections.
