# Supplementary material for: CARD-FISH in the Sequencing Era: Opening a New Universe of Protistan Ecology
Source: Front Microbiol. 2021 Mar 4;12:640066. doi: 10.3389/fmicb.2021.640066 (PMC7970053; doi:10.3389/fmicb.2021.640066)
Supplement: Supplementary File 2 — Detailed description of the recommended CARD-FISH protocol. [file Data_Sheet_2.PDF]

DETAILED CARD-FISH PROTOCOL FOR PROTISTS

STEP 1. Sample fixation with Lugol's solution-formalin-sodium thiosulphate:

STEPS FOR FIXING **100 ML** OF WATER SAMPLE (DO NOT INHALE FIXATIVES, IF POSSIBLE WORK IN FUME HOOD):

1. Add 0.5-1ml of Lugol's solution (final concentration (fc.). 0.5-1%);
2. Within max. 1 min, add 2.7-5.4 ml of methanol stabilized 37% formalin or 5-10 ml of 20% paraformaldehyde solution (fc. 1-2%);
3. Add dropwise 3% sodium thiosulphate solution using a syringe with a 0.2 µm syringe filter until the colorization from Lugol's solution completely disappears;
4. Store up to 1 h at room temperature (RT, 15-25°C) or 24 h at 4°C in the fridge.

COMMENTS

Fixation is the key step for the preservation of protistan cells for the CARD-FISH procedure. It increases cell permeability, which can be a critical factor for protists with thick and/or complex cellular structures (Ku and Sebé-Pedrós, 2019). Insufficiently fixed cells will degrade and rupture during filtration. On the other hand, harsh and highly concentrated fixatives can shrink and deform cells (Fried et al., 2002), eventually resulting in the ejection of particles ingested in their food vacuoles (Sherr et al., 1989). A frequently use fixative for CARD-FISH on protists is particle-free (filtered through 0.2 µm filter) buffered paraformaldehyde (PFA) added to the samples at 1-4% final concentration (Not et al., 2002; Mangot et al., 2009), based on a protocol developed for prokaryotes (Amann et al., 1990). However, PFA deforms and dissolves fragile HF cells (Jeuck et al., 2017), and is not suitable for food vacuole content analyses (Sherr et al., 1989). Moreover, if the samples cannot be filtered within 24 h when stored at 4°C, or within 1 h when stored at room temperature, they become over-fixed. PFA reacts with uncharged amino acids, forming cross-links that stabilize proteins. Excessive fixation makes ribosomes inaccessible to probes, resulting in weak or even lack of fluorescent signals. Preservation of samples with Lugol's solution at final concentrations of 0.5-1% before adding PFA or formalin (FA) improves the cell recovery and helps to keep their original morphology and size (Jeuck et al., 2017). It also minimizes the egestion of food vacuole contents by phagotrophic flagellates and ciliates (Sherr et al., 1989; Šimek et al., 2019). Lugol's solution can be prepared at different pH, from acidic to alkaline (Edler and Elbrachter, 2010), which allows using it for samples from extreme environments. The use of Lugol's solution as the first fixative also enables storage of samples at 4°C for about one week before the filtration, compared to 24 h when samples are fixed with PFA or formalin, which can be added once samples are about to be processed further. However, longer storage causes loss and aggregation of cells thus is not recommended (Thiele et al., 2014; Williams et al., 2015). The yellow color of Lugol's solution needs to be decolorized with a 3% sodium thiosulphate solution ($Na_2S_2O_3$) added dropwise before processing samples for CARD-FISH, because iodine is autofluorescent in blue light. Moreover, we observed that iodine in Lugol's solution may interfere with DTAF that is used for preparation of fluorescent tracers such as fluorescently labeled bacteria (FLB) in combination with CARD-FISH. Thus, if these two methods are to be combined, the fixation with Lugol's solution should not exceed 1 min. Glutaraldehyde at a final concentration of 1-2 % is often used for analyses of FLB uptake rates (Vazquez-Dominguez et al., 1999; Šimek et al., 2019), as it enhances their fluorescent signals and thus facilitates easy counting. However, we discourage its use for fixing samples for CARD-FISH. First of all, glutaraldehyde is stronger than PFA, which makes over-fixation more likely, and second, it induces green autofluorescence of the cells (Lee et al., 2013), which may compromise microscopic analysis.

STEP 2. Filtration:

PAY ATTENTION TO WHEN THE PUMP IS SWITCHED ON AND OFF, AND WHETHER THE FILTRATION SYSTEM GETS DEPRESSURIZED. PUMP **MUST BE SWITCH OFF** AND THERE **MUST BE NO PRESSURE** WHEN PLACING THE FILTERS AND POURING THE SAMPLE. WHEN PROCESSING SAMPLES FIXED WITH FORMALIN, WORK IN FUME HOOD. DISCARD THE WASTE ACCORDING TO THE LOCAL REGULATIONS CONCERNING PARAFORMALDEHYDE (CONCENTRATION IN THE WASTE IS ROUGHLY HALF OF THAT IN THE SAMPLES).

1. Immerse a support filter (cellulose esters, diameter 47 mm, pore size 1.2-2.5 µm) in DI water in a Petri dish;
2. Put the wet support filter on the filtration tower;
3. Label with sample code on the shiny side of the polycarbonate (PC, diameter 47 mm, pore size 0.6-1 µm) filter at the edge using a soft pencil;
4. Place the labelled PC filter shiny site up on the support filter on the filtration tower. It should get moist from the support filter;
5. Carefully close the filtration tower, take care that the filters do not move;
6. Mix the sample by slowly turning the bottle ca. 10-20 times up and down. Pour it gently into the filtration funnel onto the filter. The sample volume depends on the abundance of NF in the sample, which should be first assessed on 25 mm filters (e.g by DAPI staining and epi-fluorescence microscopy), and the final volume for 47 mm filter should be ca. 4x larger;
7. Switch on the pump, the vacuum underpressure should be < 200 mbar;
8. After the whole sample volume goes through, wash the filter three times with 20-30 ml of particle-free deionized (DI) water or 1xPBS;
9. Once all the water passes through, open the filtration tower;
10. Switch off the pump;
11. Remove the PC filter from the filtration tower (the support filter can be reused), place it in a clean box and let it air dry. Avoid drafty places;
12. Continue with remaining samples;
13. The dried and labelled filters may be piled up in a Petri dish. They should be separated with blue protection papers, as the PC filters in a new box;
14. Filters can be stored at -20°C or at -80°C at least for a few months to years.

COMMENTS

Filtration is the most efficient way of collecting flagellated and other protists for subsequent quantification. Effective quantitative recovery of small flagellates from sediment samples has been achieved using isopycnic centrifugation in non-linear 50% Percoll gradient at 4,300 g for 15 min, or rate zonal centrifugation at 100 g for 0.5-5 min (Starink et al., 1994). However, the efficiency of centrifugation for pelagic protists that tend to be buoyant to avoid sinking is questionable, and the method is not commonly used for water samples.

Samples for CARD-FISH should be filtered onto white polycarbonate filters that retain microbial cells on their surface. In general, cells should be collected on the shiny side of a filter, but doing otherwise does not disqualify samples from further analysis. Filters should be labeled at the edge with a sample name/number using a soft pencil. We recommend polycarbonate filters with 47 mm diameter and 0.6-1.0 µm pore size (Piwosz and Pernthaler, 2010; Morgan-Smith et al., 2013; Pernice et al., 2014). They should collect majority of protists, including Ostreococcus taurii, the smallest known eukaryote (Chrétiennot-Dinet et al., 1995; Massana, 2011), and provide a sufficient filter area that can be cut with scissors or a blade into 12-20 filter sections for application of many different probes separately. We do not recommend 0.2 µm pore size filters. The reason is that protists are about 1000 times less abundant than prokaryotes (Massana et al., 2006; Anderson et al., 2013; Sirová et al., 2018), thus larger volumes need to be filtered to collect enough cells for quantitative analysis. Filters with pore

sizes 0.6-1.0 µm enable to filter larger volumes required to collect enough protistan cells for quantitative analysis, while the vast majority of prokaryotes will pass through. In contrast, a 0.2 µm filter would accumulate large numbers of prokaryotes, creating thus a high fluorescent background and making it difficult to differentiate small eukaryotic nuclei from prokaryotes, or bacteria ingested in food vacuoles of protists from those only retained on the filter surface. This may considerably bias protistan counts or quantification of ingested bacteria. We also do not recommend small filters (diameter 25 mm), as they provide a too small area for analysis, especially if more new probes are to be tested. The filtered sample volume needs to be carefully adjusted to avoid multiple layers of cells, which would also hamper microscopic analysis and may cause detachment of cells during embedding in agarose. Cell density can be first assessed on 25 mm diameter filters (i.e. by DAPI staining and epi-fluorescence microscopy), and the final volume for 47 mm diameter filters would be ca. 4x larger. An even cell distribution on filters is maximized by (i) gently pouring the sample into a filtration funnel before using vacuum in the filtration system, and by (ii) the use of a support filter from cellulose esters with a pore size larger than the one of the polycarbonate filter. Vacuum underpressure should be kept between 150-250 mBar to avoid cell loss due to disruption (Not et al., 2002; Lepère et al., 2010). Once the whole volume of a fixed sample is filtered, the filter is 3-times carefully washed by 20-30 ml of particle free (filtered through 0.2 µm filter) PBS or deionized or distilled water to remove formalin/PFA to prevent sample overfixation, and also to wash out most of the prokaryotes yet retained on the filter surface. Then the filter is gently removed, labeled if not done so before, and air-dried (drafty places should be avoided). Dehydration in increasing concentrations of ethanol (e.g. 50, 80 and 100 % for 3 min. each) may allow for longer sample storage (Not et al., 2002), but may also lead to cells detachment and loss. Dried and labeled filters can be piled up in a Petri dish and stored at -20°C or at -80°C at least for a few months to years.

STEP 3. Embedding:

1. Warm up a 0.1% agarose solution to ca. 40°C in a microwave oven (it should be hand warm). Pour it into a 50 mm (diameter) Petri dish;
2. Place a filter upside down into the agarose, dip the edges and gently shake the Petri dish until the filter is completely covered;
3. Carefully place the filters with filtered (shiny) side facing up on a parafilm or a glass plate. Let them dry at ca. 40°C (it takes about 20-30 min);
4. Carefully remove filters, use a few drops of ethanol if needed. Filters can be stored at -20°C or at -80°C at least for a few months to years.

COMMENTS

The CARD-FISH procedure includes several sample handling steps and incubations in buffers, what may cause cell loss. This is minimized by embedding the filters in a 0.1% solution of low-melting-point agarose (Fazi et al., 2007). Agarose with a gel-point below 30°C is recommended. The use of high-melting-point agarose would require high temperatures to keep the solution liquid, which may damage cells. Higher concentrations of agarose (1% and more) might cause folding of the filters. Filters need to be dried with filtered side facing up onto parafilm, and gently removed with aid of a few drops of ethanol.

STEP 4 & STEP 5. Permeabilization of cells and Inactivation of endogenous peroxidases:

1. Pour 0.01M HCl solution into a 50 mm (diameter) Petri dish;
2. Place a filter upside down onto the HCl, dip the edges and gently shake the Petri dish until the filter is completely covered. 20-30 filters can be incubated at once;
3. Incubate for 20 min at RT;
4. Move all filters quickly to a Petri dish with 1xPBS;
5. Wash them twice in the PBS and DI water;
6. Shortly deep them in 95-100% ethanol;
7. Let them air dry;
8. Filters can be stored at -20°C or at -80°C at least for a few months to years.

COMMENTS

Additional enzymatic permeabilization is not required for hybridization of some protists (Bochdansky and Huang, 2010), and it is enough to simply dip the filters into 0.01 M HCl solution, which at the same time inactivates endogenous peroxidase that may produce false positive signals during the CARD. Some protist cells are very fragile and permeabilization should be gentle enough so that cells retain their shape and food vacuole contents, and strong enough so that probes can enter the cells. A digestion step with chitinase was tested for fungi, but it did not improve number of the hybridized cells (Jones et al., 2011). On the other hand, thick organic extracellular polymers may hamper penetration of probes and dyes into cells, as was observed for deep sea marine snow (Bochdansky et al., 2017). In such cases, incubation of filters in a 25 mM EDTA for 15 min dissolves the polymers and enables a successful CARD-FISH procedure (Cavaliere et al., 2014). An appropriate digestion with lysozyme, proteinase K or achromopetidase may be required if endosymbiotic or ingested prokaryotes are targeted (Gerea et al., 2013).

STEP 6. Hybridization:

DISCARD THE USED HYBRIDIZATION MIXTURE ACCORDING TO THE LOCAL REGULATIONS, AS IT CONTAINS FORMAMIDE, WHICH IS HAZARDOUS

CUTTING AND LABELLING THE FILTERS

1. Cut filters into 12 (angle 30°) to 20 (angle 18°) sections using clean scissors, scalpels or blades. Use a plate of medium soft plastic as a support. A drawing of a 50 mm circle divided into sections can be printed out (see examples at the end of the protocol) and glued with tape to the plate for cutting if it is transparent;
2. Label the sections at the edges with sample and probe code. A dot after the code facilitates to work out which side is up during mounting of the sections.

HYBRIDIZATION

3. Mix 300 µL of the appropriate hybridization buffer (HB) with 3 µl of the probe in a 0.5 ml tube (for few filter section), or 900 µL of HB with 9 µl of the probe (1.5 ml tube, for up to 10 sections), or 2-4 mL of HB with 20-40 µl of the probe (small (30 mm) Petri dish, tens of sections);
4. Submerge the labelled sections into the hybridization mixture, avoid folding;
5. Incubate for 3 to up to 48 h in a hybridization oven at the appropriate temperature (typically 35 or 46°C).

COMMENTS

Before starting the hybridization procedure, 47 mm diameter filters need to be cut into small triangular sections (12-20) that should be labeled at the edges with a sample and a probe code (Supplementary Figure S1). Clean scissors, scalpels or blades can be used for cutting and a soft pencil should be used for labeling. Labeled sections can be processed together. Up to 10 sections can be hybridized in a 1.5 ml centrifuge tube containing 900 µl of hybridization buffer. Larger numbers should be hybridized in 2-4 ml of hybridization buffer in a small petri dish (diameter ca. 30 mm). An experienced person can proceed even 100 samples (filters sections) at once. The hybridization buffer (Supplementary File 3) contains varying amounts of the denaturing agent formamide (Supplementary Table S2). Portions of hybridization buffers with different concentrations of formamide can be stored at -20°C and mixed with the probe immediately before hybridization (in proportion 1:100, Table 2, Supplementary Files 1 and 2). Stringency of the hybridization at a certain temperature is determined by the concentration of formamide in the hybridization buffer, and of NaCl in the washing buffer (Pernthaler et al., 2004). Therefore, it is of crucial importance that the temperature during hybridization is stable within ±0.2°C or less. Decrease in temperature by 10°C can be compensated by increasing formamide concentration by 20%. The standard duration of this step is 3 h (Not et al., 2002; Piwosz and Pernthaler, 2010), but its prolongation (even up to 4 days) may improve the signal intensity from less active cells (Lim et al., 1993; Amann and Fuchs, 2008; Morgan-Smith et al., 2013; Mukherjee et al., 2015). For instance, we use longer times (12-24 h) for hydridization of members of Kinetoplastea from freshwater lakes (Mukherjee et al., 2015; Mukherjee et al., 2019; Šimek et al.,

2020). The optimal hybridization conditions need to be carefully tested for each probe. Most probes are hybridized at 35°C, but for some the optimal stringency can be achieved only at 46°C (Simon et al., 1997; Metfies and Medlin, 2007; Piwosz, 2019); see also Table 1 and Supplementary Table S1). Despite initial concerns about the HRP enzyme inactivation at higher temperatures, it was shown to be reactive up to 57°C (Ishii et al., 2004). The composition of the hybridization buffer according to two most frequently used protocols is given in Table 2. Once the hybridization starts, the protocol must be followed to the end and the filters cannot get dried between the steps. However, in case of an unpredicted event, the hybridization step may be prolonged.

STEP 7. Washing:

1. Prepare the washing buffer (WB) in a 50 mL centrifuge tube:
    a. 500 µL of 0.5M EDTA (pH 8.0)
    b. 1000 µL of 1M Tris-HCl (pH 7.4)
    c. X µL of 5 M NaCl (see Table S2)
    d. Fill up to 50 mL to DI water
    e. 50 µL of 10% SDS
2. Pre-warm to the appropriate temperature (usually 2°C higher than the hybridization temperature). It is very important to pre-warm the WB, thus prepare it immediately after the onset of hybridization;
3. Transfer the filter sections from the hybridization mixture to WB
4. Incubate for 30 min.

COMMENTS

The washing step removes probes weakly hybridized to non-target cells and also non-hybridized. The conditions must be stringent to remove unspecifically bound probes and to retain those attached to the target sequence. The standard washing buffer contains 20 mM of Tris-HCl (pH 7.4-7.5), 5 mM EDTA (pH 8.0), 0.01% SDS and variable concentration of NaCl. The stringency is achieved with appropriate concentration of NaCl, what depends on the concentration of the formamide in the hybridization buffer and the temperature of the hybridization and washing steps (Supplementary Table S2). Washing is done at temperature 2°C higher than the hybridization, and it is important to keep the temperature stable within ±0.2°C. We usually conduct a 30 min-long washing step (Supplementary File 1), but there is a high variability of its duration in the literature (Not et al., 2002; Medina-Sánchez et al., 2005; Morgan-Smith et al., 2011; Pernice et al., 2014; Lepère et al., 2016).

STEP 8. CARD:

1. Transfer the filter sections to 0.01% PBS-T and incubate for 45 min at 37°C;
2. Prepare the amplification mixture (just before its use). If hybridization is done in a Petri dish (see STEP 6 p. 3), so should be CARD. In such a case use 2-4 times larger volumes:
    a. 1 mL of amplification buffer
    b. 10 µL of 0.15% H_2O_2
    c. 2 µL of fluorochrome-labelled tyramide solution;
3. Remove excess liquid from the filter sections by quickly dapping them on a blotting paper (e.g. paper tissues), and transfer them immediately to the amplification mixture. **DO NOT** allow sections to dry;
4. Incubate for 30 min at 37°C in the dark;
5. Remove excess liquid from the filter sections by quickly dapping them on a blotting paper, and transfer them immediately to 0.01% PBS-T. **DO NOT** allow sections to dry;
6. Incubate for 15 min at 37°C in the dark;
7. Wash filters in DI water and 95-100% ethanol at room temperature in the dark
8. Let them air dry in the dark. Avoid drafty places and breathing ;)

COMMENTS

Catalyzed reporter deposition (CARD) or tyramide signal amplification (TSA) was first applied in immunostaining assays, in which the fluorescent signals were enhanced by deposition of a large number of biotin or fluorescein labeled tyramines (Bobrow et al., 1991). As explained in Pernthaler et al. (2002), horse radish peroxidase generates highly reactive free radicals during the dimerization of phenolic compounds such as tyramines (Zaitsu and Ohkura, 1980). They subsequently bind to electron-rich moieties at or near the peroxidase binding site, introducing fluorescent molecules at the hybridization site, in result increasing the signal intensity several times. Fluorescently labeled tyramines can be purchased or synthetized in a laboratory as described in Pernthaler et al. (2004). There is a wide variety of available dyes, but those emitting green light (emission maximum at 520 nm), such as Fluorescein isothiocyanate (FITC), or Alexa488, are the most commonly used. Recently, sulfonate groups that reduce aggregation and increase signal intensity in the Alexa dyes (introduced by Molecular Probes, Inc) have been replaced with pegylation in CF® dyes (Biotum, Inc), which reduces the negative charge of the fluorescent molecules and promises to reduce the non-specific background. They have been shown to provide better results for yellow and red colors (Lehmann et al., 2015).

The amplification step needs to be preceded with equilibration of probe-delivered HPR enzymes (Pernthaler et al., 2002). This step can be done in TNT buffer (10mM Tris-HCl, 150 mM NaCl, 7% Tween 20 (Not et al., 2002)) or in PBS-T buffer (137 mM NaCl, 10 mM Na_2HPO_4, 2.7 mM KCl, 1.8 mM KH_2PO_4, 0.01% Triton-X (Piwosz and Pernthaler, 2010)). Commercial CARD/TSA kits are also available (Not et al., 2002). The composition of the amplification buffer used by us is given in Table 2 and Supplementary File 3. CARD can be done at room temperature or at 37°C or even 48°C. The efficiency of the amplification reaction increases with the temperature, but too hot conditions (55°C) may result in high background fluorescence (Ishii et al., 2004). Subsequent washing in the TNT or PBS-T buffer removes unbound tyramides and reduces background fluorescence. A final washing step in deionized water and dehydration in ethanol ends the CARD-FISH procedure.

STEP 9. Mounting:

DAPI (AS MOST DNA DYES) IS CARCINOGENIC, USE GLOVES. THERE IS NO NEED TO WORK IN A FUME HOOD. DISCARD THE WASTE AND USED MICROSCOPIC SLIDS ACCORDING TO THE LOCAL REGULATIONS.

1. Label microscopic slides with the probe and samples name at the edge. 6-8 sections fit at one slide;
2. Pipette 6-8 drops of the DAPI-mix on the slide (DAPI-mix contains glycerol and is hard to pipette precise volumes, but it does not matter);
3. Put one filter section on each drop;
4. Pipette drops of the DAPI-mix between the filter sections;
5. Gently cover the sections with the cover glass. Use high quality clean glass, do not buy cheapest one until tested;
6. Keep it in the dark for 10 minutes
7. Gently press the cover glass to remove excessive volume of the DAPI-mix and air bubbles. A pipette tip or the back of a pencil can be used carefully. Clean with a paper tissue, pay attention not to move the sections;
8. Store at -20°C in the dark. The signals are stable for about 1 year.

COMMENTS

Hybridized and dried filter pieces need to be counterstained with a DNA dye, which can be added to a glycerol containing anti-fading mixtures like Vectashield (Vector laboratories) used to mount the samples on microscopic slides and reduce fading of the signals. DAPI is the most common general nucleic acid stain (Coleman, 1980; Porter and Feig, 1980). Other possibilities include SYBR-Green and propidium iodide (Chambouvet et al., 2008). Protists with cell walls containing cellulose or chitin can be also stained with calcofluor white (Lepère et al., 2016). It is important to use DNA dyes whose excitation and emission spectra do not overlap with that of the fluorochrome used for CARD.

STEP 10. Microscopic evaluation:

1. Use an epi-fluorescent microscope with appropriate filter sets;
2. Use oil-immersion objectives with 40-100 × magnification and 10-20 × eyepiece;
3. If possible, make photographical documentation;
4. If manual counts are done, count first the probe signals, and then the DAPI signals to minimize bleaching of the signals;
5. Minimum 100 DAPI-stained NF should be counted

COMMENTS

Hybridized protistan cells are counted as percent of all protists detected with a general nucleic acid stain. Moreover, a contribution of specific lineages to abundance of the higher taxonomic group can be estimated as well. Independent total NF counts are required to estimate absolute numbers of the targeted HF and MF lineages. However, the absolute number of protists should be assessed from counts obtained with general eukaryotic probes, because nuclei of small protists can be mistaken for prokaryotes (Beardsley et al., 2005; Pernice et al., 2014). Recently, an automatic image acquisition and analysis has been developed for protists that allows for processing even up to 100 samples per day (Mangot et al., 2018).

References

Amann, R., and Fuchs, B.M. (2008). Single-cell identification in microbial communities by improved fluorescence in situ hybridization techniques. *Nature Review Microbiology* 6**,** 339-348.

Amann, R.I., Binder, B.J., Olson, R.J., Chisholm, S.W., Devereux, R., and Stahl, D.A. (1990). Combination of 16S ribosomal-RNA-targeted oligonucleotide probes with flow-cytometry for analyzing mixed microbial populations. *Applied and Environmental Microbiology* 56(6)**,** 1919-1925.

Anderson, R., Wylezich, C., Glaubitz, S., Labrenz, M., and Jurgens, K. (2013). Impact of protist grazing on a key bacterial group for biogeochemical cycling in Baltic Sea pelagic oxic/anoxic interfaces. *Environmental Microbiology* 15(5)**,** 1580-1594. doi: 10.1111/1462-2920.12078.

Beardsley, C., Knittel, K., Amann, R., and Pernthaler, J. (2005). Quantification and distinction of aplastidic and plastidic marine nanoplankton by fluorescence in situ hybridization. *Aquatic Microbial Ecology* 41**,** 163-169.

Bobrow, M.N., Shaughnessy, K.J., and Litt, G.J. (1991). Catalyzed reporter deposition, a novel method of signal amplification. II. Application to membrane immunoassays. *J Immunol Methods* 137(1)**,** 103-112. doi: 10.1016/0022-1759(91)90399-z.

Bochdansky, A., Clouse, M., and Herndl, G. (2017). Eukaryotic microbes, principally fungi and labyrinthulomycetes, dominate biomass on bathypelagic marine snow. *The ISME Journal* 11**,** 362–373. doi: 10.1038/ismej.2016.113.

Bochdansky, A.B., and Huang, L. (2010). Re-evaluation of the EUK516 probe for the domain Eukarya results in a suitable probe for the detection of kinetoplastids, an important group of parasitic and free-living flagellates. *Journal of Eukaryotic Microbiology* 57(3)**,** 229–235. doi: 10.1111/j.1550-7408.2010.00470.x.

Cavaliere, R., Ball, J.L., Turnbull, L., and Whitchurch, C.B. (2014). The biofilm matrix destabilizers, EDTA and DNaseI, enhance the susceptibility of nontypeable *Hemophilus*

influenzae biofilms to treatment with ampicillin and ciprofloxacin. *MicrobiologyOpen* 3(4)**,** 557-567. doi: 10.1002/mbo3.187.

Chambouvet, A., Morin, P., Marie, D., and Guillou, L. (2008). Control of toxic marine dinoflagellates blooms by serial parasitic killers. *Science* 322**,** 1254-1257.

Chrétiennot-Dinet, M.J., Courties, C., Vaquer, A., Neveux, J., Claustre, H., Lautier, J., et al. (1995). A new marine picoeucaryote: *Ostreococcus tauri* gen. et sp. nov. (Chlorophyta, Prasinophyceae). *Phycologia* 34(4)**,** 285-292. doi: 10.2216/i0031-8884-34-4-285.1.

Coleman, A.W. (1980). Enhanced detection of bacteria in natural environments by fluorochrone staining of DNA. *Limnology and Oceanography* 25(5)**,** 948-951.

Edler, L., and Elbrachter, M. (2010). "The Uthermohl method for quantitative phytoplankton analysis," in *Microscopic and molecular methods for quantitative phytoplankton analysis,* eds. B. Karlson, C. Cusack & E. Bresnan. (Paris: UNESCO), 13-20.

Fazi, S., Amalfitano, S., Pizzetti, I., and Pernthaler, J. (2007). Efficiency of fluorescence in situ hybridization for bacterial cell identification in temporary river sediments with contrasting water content. *Systematic and Applied Microbiology* 30(6)**,** 463-470.

Fried, J., Ludwig, W., Psenner, R., and Schleifer, K.H. (2002). Improvement of ciliate identification and quantification: a new protocol for fluorescence *in situ* hybridization (FISH) in combination with silver stain techniques. *Systematic and Applied Microbiology* 25(4)**,** 555-571. doi: 10.1078/07232020260517706.

Gerea, M., Queimaliños, C., Schiaffino, M.R., Izaguirre, I., Forn, I., Massana, R., et al. (2013). *In situ* prey selection of mixotrophic and heterotrophic flagellates in Antarctic oligotrophic lakes: An analysis of the digestive vacuole content. *Journal of Plankton Research* 35(1)**,** 201–212. doi: 10.1093/plankt/fbs085.

Ishii, K., Mussmann, M., MacGregor, B., and Amann, R. (2004). An improved fluorescence *in situ* hybridization protocol for the identification of bacteria and archaea in marine sediments. *FEMS microbiology ecology* 50**,** 203-213. doi: 10.1016/j.femsec.2004.06.015.

Jeuck, A., Nitsche, F., Wylezich, C., Wirth, O., Bergfeld, T., Brutscher, F., et al. (2017). A comparison of methods to analyze aquatic heterotrophic flagellates of different taxonomic groups. *Protist* 168(4)**,** 375-391. doi: 10.1016/j.protis.2017.04.003.

Jones, M.D.M., Forn, I., Gadelha, C., Egan, M.J., Bass, D., Massana, R., et al. (2011). Discovery of novel intermediate forms redefines the fungal tree of life. *Nature* 474(7350)**,** 200-203. doi: 10.1038/nature09984.

Ku, C., and Sebé-Pedrós, A. (2019). Using single-cell transcriptomics to understand functional states and interactions in microbial eukaryotes. *Philosophical Transactions of the Royal Society B: Biological Sciences* 374(1786)**,** 20190098. doi: doi:10.1098/rstb.2019.0098.

Lee, K., Choi, S., Yang, C., Wu, H.-C., and Yu, J. (2013). Autofluorescence generation and elimination: A lesson from glutaraldehyde. *Chemical Communications* 49**,** 3028-3030. doi: 10.1039/c3cc40799c.

Lehmann, M., Lichtner, G., Klenz, H., and Schmoranzer, J. (2015). Novel organic dyes for multicolor localization-based super-resolution microscopy. *Journal of biophotonics* 9. doi: 10.1002/jbio.201500119.

Lepère, C., Masquelier, S., Mangot, J.-F., Debroas, D., and Domaizon, I. (2010). Vertical structure of small eukaryotes in three lakes that differ by their trophic status: a quantitative approach. *The ISME Journal* 4(12)**,** 1509-1519. doi: 10.1038/ismej.2010.83.

Lepère, C., Ostrowski, M., Hartmann, M., Zubkov, M.V., and Scanlan, D.J. (2016). In situ associations between marine photosynthetic picoeukaryotes and potential parasites - a role for fungi? *Environmental Microbiology Reports* 8(4)**,** 445-451. doi: 10.1111/1758-2229.12339.

Lim, E.L., Amaral, L.A., Caron, D.A., and DeLong, E.F. (1993). Application of rRNA-based probes for observing marine nanoplanktonic protists. *Applied and Environmental Microbiology* 59(5)**,** 1647-1655.

Mangot, J.-F., Forn, I., Obiol, A., and Massana, R. (2018). Constant abundances of ubiquitous uncultured protists in the open sea assessed by automated microscopy. *Environmental Microbiology* 20(10)**,** 3876-3889. doi: 10.1111/1462-2920.14408.

Mangot, J.F., Lepere, C., Bouvier, C., Debroas, D., and Domaizon, I. (2009). Community structure and dynamics of small eukaryotes targeted by new oligonucleotide probes: new insight into the lacustrine microbial food web. *Applied and Environmental Microbiology* 75(19)**,** 6373-6381.

Massana, R. (2011). "Eukaryotic picoplankton in surface oceans," in *Annual Review of Microbiology,* eds. S. Gottesman & C.S. Harwood.), 91-110.

Massana, R., Guillou, L., Terrado, R., Forn, I., and Pedros-Alio, C. (2006). Growth of uncultured heterotrophic flagellates in unamended seawater incubations. *Aquatic Microb Ecol* 45**,** 171-180.

Medina-Sánchez, J.M., Felip, M., and Casamayor, E.O. (2005). Catalyzed reported deposition-fluorescence *in situ* hybridization protocol to evaluate phagotrophy in mixotrophic protists. *Applied and Environmental Microbiology* 71(11)**,** 7321-7326. doi: 10.1128/aem.71.11.7321-7326.2005.

Metfies, K., and Medlin, L. (2007). Refining cryptophyte identification with DNA-microarrays. *Journal of Plankton Research* 12**,** 1071-1075.

Morgan-Smith, D., Clouse, M.A., Herndl, G.J., and Bochdansky, A.B. (2013). Diversity and distribution of microbial eukaryotes in the deep tropical and subtropical North Atlantic Ocean. *Deep Sea Research Part I: Oceanographic Research Papers* 78**,** 58-69. doi: 10.1016/j.dsr.2013.04.010.

Morgan-Smith, D., Herndl, G.J., van Aken, H.M., and Bochdansky, A.B. (2011). Abundance of eukaryotic microbes in the deep subtropical North Atlantic. *Aquatic Microbial Ecology* 65(2)**,** 103-115. doi: 10.3354/ame01536.

Mukherjee, I., Hodoki, Y., and Nakano, S.-i. (2015). Kinetoplastid flagellates overlooked by universal primers dominate in the oxygenated hypolimnion of Lake Biwa, Japan. *Fems Microbiology Ecology* 91(8). doi: 10.1093/femsec/fiv083.

Mukherjee, I., Hodoki, Y., Okazaki, Y., Fujinaga, S., Ohbayashi, K., and Nakano, S.-i. (2019). Widespread Dominance of Kinetoplastids and Unexpected Presence of Diplonemids in Deep Freshwater Lakes. *Frontiers in Microbiology* 10**,** 2375. doi: 10.3389/fmicb.2019.02375.

Not, F., Simon, N., Biegala, I.C., and Vaulot, D. (2002). Application of fluorescent in situ hybridization coupled with tyramide signal amplification (FISH-TSA) to assess eukaryotic picoplankton composition. *Aquatic Microbial Ecology* 28(2)**,** 157-166. doi: 10.3354/ame028157.

Pernice, M.C., Forn, I., Gomes, A., Lara, E., Alonso-Sáez, L., Arrieta, J.M., et al. (2014). Global abundance of planktonic heterotrophic protists in the deep ocean. *The ISME Journal* 9(3)**,** 782-792. doi: 10.1038/ismej.2014.168.

Pernthaler, A., Pernthaler, J., and Amann, R. (2002). Fluorescence *in situ* hybridization and catalyzed reporter deposition for the identification of marine bacteria. *Appl Environ Microbiol* 68(6)**,** 3094-3101.

Pernthaler, A., Pernthaler, J., and Amann, R. (2004). Sensitive multi-color fluorescence in situ hybridization for the identification of environmental microorganisms. *Molecular Microbial Ecology Manual* 3(11)**,** 711-726.

Piwosz, K. (2019). Weekly dynamics of abundance and size structure of specific nanophytoplankton lineages in coastal waters (Baltic Sea). *Limnology and Oceanography* 64(5)**,** 2172-2186. doi: 10.1002/lno.11177.

Piwosz, K., and Pernthaler, J. (2010). Seasonal population dynamics and trophic role of planktonic nanoflagellates in coastal surface waters of the Southern Baltic Sea. *Environmental Microbiology* 12(2)**,** 364-377.

Porter, K.G., and Feig, Y.S. (1980). The use of DAPI for identifying and counting aquatic bacteria. *Limnology and Oceanography* 25(5)**,** 943-948.

Sherr, B.F., Sherr, E.B., and Pedros-Alio, C. (1989). Simultaneous measurement of bacterio-plankton production and protozoan bacterivory in estuarine water. *Marine Ecology-Progress Series* 54**,** 209-219.

Šimek, K., Grujčić, V., Mukherjee, I., Kasalický, V., Nedoma, J., Posch, T., et al. (2020). Cascading effects in freshwater microbial food webs by predatory Cercozoa, Katablepharidacea and ciliates feeding on aplastidic bacterivorous cryptophytes. *FEMS Microbiology Ecology* In press.

Šimek, K., Grujčić, V., Nedoma, J., Jezberová, J., Šorf, M., Matoušů, A., et al. (2019). Microbial food webs in hypertrophic fishponds: Omnivorous ciliate taxa are major protistan bacterivores. *Limnology and Oceanography* 64(5)**,** 2295-2309. doi: 10.1002/lno.11260.

Simon, N., Brenner, J., Edvardsen, B., and Medlin, L.K. (1997). The identification of *Chrysochromulina* and *Prymnesium* species (Haptophyta, Prymnesiophyceae) using

fluorescent or chemiluminescent oligonucleotide probes: a means for improving studies on toxic algae. *European Journal of Phycology* 32(4)**,** 393-401.

Sirová, D., Bárta, J., Šimek, K., Posch, T., Pech, J., Stone, J., et al. (2018). Hunters or farmers? Microbiome characteristics help elucidate the diet composition in an aquatic carnivorous plant. *Microbiome* 6(1)**,** 225. doi: 10.1186/s40168-018-0600-7.

Starink, M., Bär-Gilissen, M.J., Bak, R.P., and Cappenberg, T.E. (1994). Quantitative centrifugation to extract benthic protozoa from freshwater sediments. *Applied and environmental microbiology* 60(1)**,** 167-173. doi: 10.1128/AEM.60.1.167-173.1994.

Thiele, S., Wolf, C., Schulz, I.K., Assmy, P., Metfies, K., and Fuchs, B.M. (2014). Stable composition of the nano- and picoplankton community during the ocean iron fertilization experiment LOHAFEX. *Plos One* 9(11)**,** e113244. doi: 10.1371/journal.pone.0113244.

Vazquez-Dominguez, E., Peters, F., Gasol, J.M., and Vagué, D. (1999). Measuring the grazing losses of picoplankton: methodological improvements in the use of fluorescently labeled tracers combined with flow cytometry. *Aquatic Microbial Ecology* 20(2)**,** 119-128.

Williams, O., Beckett, R., and Maxwell, D. (2015). Marine phytoplankton preservation with Lugol's: a comparison of solutions. *Journal of Applied Phycology* 28. doi: 10.1007/s10811-015-0704-4.

Zaitsu, K., and Ohkura, Y. (1980). New fluorogenic substrates for horseradish peroxidase: Rapid and sensitive assays for hydrogen peroxide and the peroxidase. *Analytical Biochemistry* 109(1)**,** 109-113. doi: 10.1016/0003-2697(80)90017-2.
