# Supplementary material for: CARD-FISH in the Sequencing Era: Opening a New Universe of Protistan Ecology
Source: Front Microbiol. 2021 Mar 4;12:640066. doi: 10.3389/fmicb.2021.640066 (PMC7970053; doi:10.3389/fmicb.2021.640066)
Supplement: Supplementary File 3 — Single page versions of the recommended CARD-FISH and double CARD-FISH protocols. [file Data_Sheet_3.PDF]

CARD–FISH PROTOCOL

FIXATION & FILTRATION:

- Fix sample with Lugol's solution (0.5%, 1 min), formaldehyde or PFA (1-2%, 1 h at room temperature (RT) or 24 h at 4°C), and add dropwise a 3% $Na_2S_2O_3$ to remove color
- Filter sample onto white polycarbonate filters (0.6-0.8 µm pore size, supporting filter, 1.2-5 µm pore size), rinse with DI H_2O or PBS, let air dry, store at -20°C or -80°C

EMBEDDING, PERMEABILIZATION & INACTIVATION PEROXIDASES:

- Melt 0.1 % agarose in microwave oven, let it cool down to ~40°C
- Dip filters in agarose, put them on parafilm or a glass plate (cells facing up), let filters dry at 30-40°C
- Remove filters from parafilm (add a drop of ethanol, peel them off carefully)
- Air dry filters and store them at -20°C (or go on with the protocol)
- Incubate filters in 0.01 M HCl for 20 min at RT
- Wash filters in PBS, DI H_2O and EtOH (at RT), let them air dry and store at -20°C (or go on with the protocol)

HYBRIDIZATION, WASHING & CARD:

- Cut filters into sections, label them with a pencil
- Prepare probe mix: 300 µl of hybridization buffer (HB) + 3 µl of probe (0.5 ml tube); 900 µl HB + 9 µl probe (1.5 ml tube) or 2-4 ml HB + 20-40 µl probe (small Perti dish) if you have more filters
- Hybridize filter sections for 3-48 h at 35/46°C
- Prepare washing buffer:
  - 500 µl 0.5 M EDTA (pH 8)
  - 1000 µl 1 M Tris/HCl (pH 7,4)
  - x µl 5 M NaCl (Table S2)
  - fill up to 50 ml with DI H_2O
  - 50 µl SDS
- Preheat washing buffer at 37/48°C
- Wash filters in washing buffer for 20-30 min at 37/48°C
- Incubate filters in PBS-T for 45 min at 37°C
- Prepare 0.15% H_2O_2:
  - 1000 µl 1 x PBS
  - 5 µl 30% H_2O_2
- Prepare amplification mix:
  - 1 ml amplification buffer
  - 10 µl 0.15% H_2O_2
  - 1-2 µl fluorescently labelled tyramide
- Dab filters onto blotting paper (e.g. paper tissue) to remove excess liquid but do not let filters run dry
- Incubate filters in tyramide solution for 30 min at 37°C in the dark
- Dab filters onto blotting paper to remove excess liquid but do not let filters run dry
- Incubate filters in PBST for 15 min at RT in the dark
- Wash filters in DI H_2O and EtOH at RT in dimmed light
- Let filters air dry in the dark
- Embed filters in DAPI-MIX or store them at -20°C

DOUBLE HYBRIDIZATION

After hybridization and CARD with the first probe filters can be stored at –20°C or immediately hybridized with the second probe. It is also possible to use filter sections that are already embedded in DAPI-MIX, you just have to remove the cover slip and wash filters in excessive EtOH and MQ to remove all oil. **The second probe must require higher percentage of formamide in the HB.**

- Incubate filter sections in 0.01 M HCl for 20 min at RT
- Mix hybridization buffer (with **higher** formamide percentage) and the second probe
- Hybridize filter sections for 3-48 h at 35/46°C
- Prepare washing buffer:
  500 µl 0.5 M EDTA (pH 8)
  1000 µl 1 M Tris/HCl (pH 7,4)
  x µl 5 M NaCl (Table S2)
  fill up to 50 ml with DI H_2O
  50 µl SDS
- Preheat washing buffer at 37/48°C
- Wash filters in washing buffer for 20-30 min at 37/48°C
- Incubate filters in PBS-T for 45 min at 37°C
- Prepare 0.15% H_2O_2:
  1000 µl 1 x PBS (or MQ)
  5 µl 30% H_2O_2
- Prepare amplification mix:
  1 ml amplification buffer
  10 µl 0,15% H2O2
  1-2 µl fluorescently labelled tyramide

  Here, a second fluorochrome must be used, e.g. *Alexa488* for the first hybrid-dization & *Alexa546* for the second
- Dab filters onto blotting paper to remove excess liquid but do not let filters run dry
- Incubate filters in tyramide solution for 30 min at 37°C in the dark
- Dab filters onto blotting paper to remove excess liquid but do not let filters run dry
- Incubate filters in PBST for 15 min at RT in the dark
- Wash filters in DI H_2O and EtOH @ RT in the dark
- Let filters air dry in the dark
- Embed filters in DAPI-MIX or store them @ -20°C
