# Supplementary material for: CARD-FISH in the Sequencing Era: Opening a New Universe of Protistan Ecology
Source: Front Microbiol. 2021 Mar 4;12:640066. doi: 10.3389/fmicb.2021.640066 (PMC7970053; doi:10.3389/fmicb.2021.640066)
Supplement: Supplementary File 4 — Recipes for all buffer, solutions and reagents used during CARD-FISH procedure. [file Data_Sheet_4.PDF]

RECIPES FOR BUFFERS AND SOLUTIONS

MOST OF THE BUFFERS AND SOLUTION CAN BE PURCHASED AS READY-TO-USE SOLUTIONS

STEP 1. Sample fixation with Lugol's solution-formalin-sodium thiosulphate:

IT IS ADVISABLE TO HAVE DEDICATED LABORATORY WARE (BOTTLES, SPOONS, MAGNETIC BARS, pH ELECTRODE, etc.) FOR PREPARATION OF FIXATIVES.

1. **Acid Lugol's solution (200 mL)**
   PREPARE IN A FUME HOOD
   1. 200 mL distilled or deionized water;
   2. 20 g potassium iodide (KI);
   3. 10 g resublimated iodine (I_2);
   4. 20 mL glacial acetic acid (concentrated CH_3COOH);

   Mix in the order listed. Make sure the previous ingredient has dissolved completely before adding the next. Store in a tightly stoppered glass bottle in cool and dark.

2. **Neutral Lugol's solution (200 mL)**
   PREPARE IN A FUME HOOD
   1. 200 mL distilled or deionized water;
   2. 20 g potassium iodide (KI);
   3. 10 g resublimated iodine (I_2);

   Mix in the order listed. Make sure the previous ingredient has dissolved completely before adding the next. Store in a tightly stoppered glass bottle in cool and dark.

3. **Alkaline Lugol's solution (200 mL)**
   PREPARE IN A FUME HOOD
   1. 100 mL distilled or deionized water;
   2. 20 g potassium iodide (KI);
   3. 10 g resublimated iodine (I_2)
   4. 50 g anhydrous sodium acetate (CH_3COONa) dissolved in 100 of water.

   Mix in the order listed. Make sure the previous ingredient has dissolved completely before adding the next. Store in a tightly stoppered glass bottle in cool and dark.

4. **20% paraformaldehyde (PFA) solution (100 mL)**
   PREPARE IN A FUME HOOD. IT IS THE BEST TO USE FRESHLY PREPARED SOLUTION
   1. Heat 75 mL of distilled or deionized water or 1xPBS to 60°C;
   2. Add 20 g PFA, cover the vessel. Dissolution is quite slow and it is accelerated by adding few drops of concentrated NaOH and mixing (e.g. using a magnetic stirrer). Alternatively, commercialized mineral water of pH close to those of the sample may help to dissolved PFA powder;
   3. Add 10 mL of 10xPBS (if PFA was dissolved in water);
   4. Cool down to 4°C

5. Adjust pH to 7.2;
6. Add distilled or deionized water to the final volume of 100 mL;
7. Filter sterile;
8. Solution can be stored at -20°C for longer time, or at 4°C for several days. When melting a frozen aliquot, make sure that the solution is completely clear and transparent (like water) before using.

5. 3% $Na_2S_2O_3$ (sodium thiosulphate) solution (100 mL)

1. Dissolve 4.7 g of $Na_2S_2O_3 \cdot x\ 5H_2O$ in 75 mL of distilled or deionized;
2. Add distilled or deionized water to the final volume of 100 mL;
3. Filter sterile;
4. Solution can be stored at 4°C.

STEP 2. Filtration:

1. 10xPBS (500 mL)

1. 40 g NaCl;
2. 1 g KCl;
3. 7.2 g Na_2HPO_4;
4. 1.2 g KH_2PO_4;
5. Dissolve in 350 mL of distilled or deionized water;
6. Adjust pH to 7.4-7.6;
7. Adjust final volume to 500 mL;
8. Autoclave;
9. Store at RT;

2. 1xPBS (1000 mL)

1. 8 g NaCl;
2. 0.2 g KCl;
3. 1.44 g Na_2HPO_4;
4. 0.24 g KH_2PO_4;
5. Dissolve in 800 mL of distilled or deionized water;
6. Adjust pH to 7.4-7.6;
7. Adjust final volume to 1000 mL;
8. Autoclave;
9. Store at RT;

OR

1. Mix 100 mL of 10x PBS with 900 mL of distilled or deionized water;
2. Autoclave;
3. Store at RT;

STEP 3. Embedding:

1. 0.1% agarose (200 mL)

1. 0.2 g low melting agarose;
2. 200 mL of distilled or deionized water;
3. Heat in microwave oven until agarose dissolves completely;
4. Store at RT, melt in microwave oven before use.

STEP 4 & STEP 5. Permeabilization of cells and Inactivation of endogenous peroxidases:

1. **0.1 M HCl (200 mL)**
   PREPARE IN A FUME HOOD. WEAR GLOVES, LABCOAT AND FACE PROTECTION
   1. Measure 183.5 mL of distilled or deionized water. Keep on ice;
   2. Carefully add 16.5 mL of 37% HCl;
   3. Store at RT

STEP 6. Hybridization:

1. **Hybridization buffer (20 mL)**
   1. 3.6 mL of 5M NaCl;
   2. 400 µL of 1M Tris-HCl (pH 7.4);
   3. 2 g dextran sulphate;
   4. **X** mL of distilled or deionized water (see table S2);
   5. Stir and heat (ca. 40°C) until dextran sulphate completely dissolves;
   6. Cool down to 4°C;
   7. Add **X** mL of formamide (see table S2);
   8. Add 2 mL of 10% blocking reagent;
   9. Add 20 µL of 10% SDS;
   10. Aliquote to 2 mL tubes;
   11. Store at -20°C.
2. **5M NaCl solution (500 mL)**
   1. Dissolve 146.1 g of NaCl in 400 mL of distilled or deionized water;
   2. Adjust final volume to 500 mL;
   3. Autoclave;
   4. Store at RT.
3. **1M 2-Amino-2-(hydroxymethyl)propane-1,3-diol (Tris)-HCl (pH 7.4) solution (500 mL)**
   1. Dissolve 78.8 g of Tris-HCl in 400 mL of distilled or deionized water;
   2. Adjust pH to 7.4;
   3. Adjust final volume to 500 mL;
   4. Autoclave;
   5. Store at RT.
4. **10% Blocking reagent**
   1. 0.438 g of NaCl;
   2. 0.5805 g maleic acid;
   3. Dissolve in 45 mL of distilled or deionized water;
   4. Adjust pH to 7.5;
   5. Adjust final volume to 50 mL
   6. Sterile filter;
   7. Add 5 g of blocking reagent;
   8. Stir and heat (ca. 40°C) until completely dissolves;
   9. Aliquote to 2 mL tubes;
   10. Store at -20°C.

5. **10% Sodium dodecyl sulphate (SDS) solution (50 mL)**
    1. Heat 40 mL of distilled or deionized water to ca. 40°C;
    2. Dissolve 5 g of SDS;
    3. Adjust final volume to 50 mL;
    4. Filter sterile;
    5. Store at RT. Do NOT autoclave or store in a fridge.

STEP 7. Washing:

1. **Washing buffer (50 mL)**
    1. 500 µL of 0.5M EDTA (pH 8.0);
    2. 1 mL of 1M Tris-HCl (pH 7.4);
    3. X µL 5 M NaCl (see Table S2);
    4. Fill up to 50 mL to DI water;
    5. 50 µL of 10% SDS;
2. **0.5 M ethylenediaminetetraacetic acid (EDTA, 500 mL)**
    1. Add 14.61 g of anhydrous EDTA to 400 mL of distilled or deionized water;
    2. Keep the vessel on the magnetic stirrer with magnetic pellet. Keep the pH probe to monitor pH (EDTA will not dissolve at pH > 8.0);
    3. Add ca. 15 g of NaOH pellets;
    4. Allow the NaOH pellets to dissolve completely, check pH;
    5. If required (pH < 8.0), add pellets one by one until pH is 8.0 or slightly higher. Be patient not to overshoot;
    6. Once EDTA dissolves completely, adjust pH to 8.0;
    7. Adjust final volume to 500 mL;
    8. Autoclave
    9. Store at RT.

STEP 8. CARD – solution and buffers:

1. **0.01% PBS-T (500 mL)**
    1. 500 mL of 1x PBS;
    2. 500 µL of 10% Triton X;
    3. Store at RT, do NOT autoclave.
2. **Amplification buffer (40 mL)**
    1. 4 mL 10x PBS;
    2. 16 mL of 5 M NaCl;
    3. 4 g dextran sulphate;
    4. Stir and heat (ca. 40°C) until dextran sulphate completely dissolves;
    5. Cool down to 4°C;
    6. Add 0.4 mL of 10% blocking reagent;
    7. Adjust final volume to 40 mL;
    8. Store at 4°C for up to 2 months.
3. **0.15% H_2O_2 (1 mL)**
    1. 1 mL of 1XPBS or distilled or deionized water;
    2. 5% of 30% H_2O_2;
    3. Prepare fresh.

STEP 8. CARD – tyramide synthesis:

1. **Tyramide stock**
   1. 1 mL dimethylformamide;
   2. 10 µl triethylamine;
   3. 10 mg Tyramine-HCl.
2. **Succinimidyl ester**
   ESTERS ARE LIGHT SENSITIVE AND PRONE TO HYDROLYSIS. THEREFORE PREPARE SHORTLY BEFORE TYRAMIDES SYNTHESIS! COOL ESTERS ON ICE UNTIL THEY ARE USED FOR SYNTHESIS.
   1. 1 mg of active fluorochrome ester;
   2. 100 µl dimethylformamide (DMF).
3. **Synthesis**
   1. Mix **succinimidyl ester** with tyramide stock in the following proportions:
      a. 100 µl of **Alexa488** + 25.2 µl of Tyramide stock;
      b. 100 µl of **Alexa546** + 14.7 µl of Tyramide stock;
      c. 100 µl of **Alexa633** + 13.1 µl of Tyramide stock;
      d. 500 µl of **Alexa350** (1 mg of active ester + 500 µL DMF) + 193 µl of Tyramide stock;
      e. 10 ml of **Carbofluorescein** (100 mg of active ester + 10 mL DMF) + 3.3 ml of Tyramide stock;
   2. Incubate at RT in the dark for about 12 h
   3. Dilute with absolute ethanol to 1 mL (Alexa488, Alexa546 or Alexa633), or to 5 mL (Alexa350);
   4. Make 50 µL aliquots and store at -20°C (stable for at least 1 year)
   5. For long term storage dessicate the aliquots in a freeze dryer or under vacuum at RT (stable for years at -20°C);
   6. For use, reconstitute desiccated tyramides with 50 µl DMF containing 20 mg mL^{-1} p-iodophenylboric acid (IPBA). IPBA will enhance the CARD-FISH signal. CAUTION: Alexa350-labeled tyramides should be dissolved in MQ and stored in the fridge; tyramides dissolved in DMF can be stored in the freezer.

STEP 9. Mounting:

1. **4′,6-diamidino-2-phenylindole (DAPI) stock (100 µg ml^{-1})**
   1. Dissolve 1 mg of DAPI in 10 mL of distilled or deionized water;
   2. Store at -20°C or 4°C (2-3 weeks) in the dark.
2. **DAPI-mix (1 µg ml^{-1})**
   1. 5 mL of 100% glycerol;
   2. 1 mL of Vectashield;
   3. 1 mL of PBS;
   4. 70 µL of DAPI-stock;
   5. Aliquot to 1.5-2 mL tubes
   6. Store at 4°C.
