# Supplementary material for: CARD-FISH in the Sequencing Era: Opening a New Universe of Protistan Ecology
Source: Front Microbiol. 2021 Mar 4;12:640066. doi: 10.3389/fmicb.2021.640066 (PMC7970053; doi:10.3389/fmicb.2021.640066)
Supplement: Supplementary File 5 — Troubleshooting guide. [file Data_Sheet_5.PDF]

TROUBLESHOOTING GUIDE

Unsuccessful CARD-FISH procedure can be identified by weak or lack of signals, high background fluorescence, and unspecific probe binding that can be recognized by diverse shape and size variability of hybridized cells, especially in case of very specific probes. Below we provide a short troubleshooting guide on how to address those problems.

Problem: weak or no signals

Possible causes	*Suggested solutions*
Overfixation	• make sure fixation does not exceed 1 h at room temperature (~25°C) or 24 h at 4°C;<br>• use lower concentration of FA/PFA;
Too high temperature during hybridization/ washing steps	• check the temperature settings;<br>• check the temperature during hybridization with an independent thermometer;<br>• repeat optimization of the hybridization conditions;
Poorly designed probe	• check secondary RNA structure whether the binding site is not in a hairpin structure. If so, design helpers;<br>• try to design another probe with a different binding site;<br>• confirm that the sequence of the ordered probe is not actually the target rRNA sequence to which the probe should bind. If so, order the correct probe in reverse-complement;
Filters dried during the procedure	• redo CARD-FISH without allowing it to happen;
Low activity or detachment of the HRP enzyme	• take a new probe aliquot from the freezer;<br>• once the probe is thawed, store it in a fridge (stable for several months) and do not freeze the aliquot again;<br>• order new probe;
Low ribosome content in the targeted groups	• prolong the hybridization step (up to 48 h);
Wrong filter set used to visualize the signals	• make sure that the chosen filter set is the right one for the used fluorescence dye

Problem: high background fluorescence

Possible causes	*Suggested solutions*
Presence of active endogenous peroxidases	• prolong inactivation step;

	• try higher concentrations of HCl;
Too much probe	• lower probe concentration in the hybridization buffer;
Too much tyramides	• lower tyramide concentration in the amplification buffer;<br>• prolong washing after the CARD step; repeat the washing step several times;<br>• add 10 min. washing step in 95-99% ethanol;

Problem: variable morphotypes from unspecific signals

Possible causes	***Suggested solutions***
Unspecific probe	• check the probe specificity in SILVA database;<br>• check the monophyly of the targeted lineage<br>• design a specific probe
Too low concentration of formamide in the hybridization buffer	• make sure a correct buffer was used;<br>• repeat optimization of the hybridization conditions;
Too high concentration of NaCl in the washing buffer	• make sure a correct buffer was used;<br>• repeat optimization of the hybridization conditions;
Too low temperature during hybridization/ washing steps	• check the temperature settings;<br>• check the temperature during hybridization with an independent thermometer;<br>• repeat optimization of the hybridization conditions;
