# Supplementary material for: CARD-FISH in the Sequencing Era: Opening a New Universe of Protistan Ecology
Source: Front Microbiol. 2021 Mar 4;12:640066. doi: 10.3389/fmicb.2021.640066 (PMC7970053; doi:10.3389/fmicb.2021.640066)
Supplement: Supplementary File 6 — Reference list for Supplementary Table 1. [file Data_Sheet_6.PDF]

References to Supplementary Table 1.

1. Giovannoni SJ, DeLong EF, Olsen GJ, Pace NR. 1988. Phylogenetic group-specific oligodeoxynucleotide probes for identification of single microbial cells. The Journal of Bacteriology 170:720-726.

2. Amann RI, Binder BJ, Olson RJ, Chisholm SW, Devereux R, Stahl DA. 1990. Combination of 16S ribosomal-RNA-targeted oligonucleotide probes with flow-cytometry for analyzing mixed microbial populations. Appl Environ Microbiol 56:1919-1925.

3. Sogin ML, Gunderson JH. 1987. Structural diversity of eukaryotic small subunit ribosomal RNAs. Evolutionary implications. Ann N Y Acad Sci 503:125-39.

4. Piwosz K, Pernthaler J. 2010. Seasonal population dynamics and trophic role of planktonic nanoflagellates in coastal surface waters of the Southern Baltic Sea. Environ Microbiol 12:364-377.

5. Rice J, Oconnor CD, Sleigh MA, Burkill PH, Giles IG, Zubkov MV. 1997. Fluorescent oligonucleotide rDNA probes that specifically bind to a common nanoflagellate, Paraphysomonas vestita. Microbiology 143:1717-1727.

6. Rice J, Sleigh MA, Burkill PH, Tarran GA, O'connor CD, Zubkov MV. 1997. Flow cytometric analysis of characteristics of hybridization of species-specific fluorescent oligonucleotide probes to rRNA of marine nanoflagellates. Appl Environ Microbiol 63:938-944.

7. Bochdansky AB, Huang L. 2010. Re-evaluation of the EUK516 probe for the domain Eukarya results in a suitable probe for the detection of kinetoplastids, an important group of parasitic and free-living flagellates. J Eukaryot Microbiol 57:229–235.

8. Edgcomb VP, Orsi W, Breiner HW, Stock A, Filker S, Yakimov MM, Stoeck T. 2011. Novel active kinetoplastids associated with hypersaline anoxic basins in the Eastern Mediterranean deep-sea. Deep Sea Research Part I: Oceanographic Research Papers 58:1040-1048.

9. Bochdansky A, Clouse M, Herndl G. 2017. Eukaryotic microbes, principally fungi and labyrinthulomycetes, dominate biomass on bathypelagic marine snow. The ISME Journal 11:362–373.

10. Morgan-Smith D, Clouse MA, Herndl GJ, Bochdansky AB. 2013. Diversity and distribution of microbial eukaryotes in the deep tropical and subtropical North Atlantic Ocean. Deep Sea Research Part I: Oceanographic Research Papers 78:58-69.

11. Lange M, Guillou L, Vaulot D, Simon N, Amann RI, Ludwig W, Medlin LK. 1996. Identification of the class prymnesiophyceae and the genus Phaeocystis with ribosomal RNA-targeted nucleic acid probes detected by flow cytometry. J Phycol 32:858-868.

12. Simon N, Campbell L, Ornolfsdottir E, Groben R, Guillou L, Lange M, Medlin LK. 2000. Oligonucleotide probes for the identification of three algal groups by dot blot and fluorescent whole-cell hybridization. J Eukaryot Microbiol 47:76-84.

13. John U, Cembella A, Hummert C, Elbrächter M, Groben R, Medlin L. 2003. Discrimination of the toxigenic dinoflagellates Alexandrium tamarense and A. ostenfeldii in co-occurring natural populations from Scottish coastal waters. Eur J Phycol 38:25-40.

14. Thiele S, Wolf C, Schulz IK, Assmy P, Metfies K, Fuchs BM. 2014. Stable composition of the nano- and picoplankton community during the ocean iron fertilization experiment LOHAFEX. Plos One 9:e113244.

15. Chambouvet A, Morin P, Marie D, Guillou L. 2008. Control of toxic marine dinoflagellates blooms by serial parasitic killers. Science 322:1254-1257.

16. Mangot JF, Lepere C, Bouvier C, Debroas D, Domaizon I. 2009. Community structure and dynamics of small eukaryotes targeted by new oligonucleotide probes: new insight into the lacustrine microbial food web. Appl Environ Microbiol 75:6373-6381.

17. Stokes NA, Calvo LMR, Reece KS, Burreson EM. 2002. Molecular diagnostics, field validation, and phylogenetic analysis of Quahog Parasite Unknown (QPX), a pathogen of the hard clam Mercenaria mercenaria. Dis Aquat Org 52:233-247.

18. Simon N, LeBot N, Marie D, Partensky F, Vaulot D. 1995. Fluorescent in situ hybridization with rRNA-targeted oligonucleotide probes to identify small phytoplankton by flow cytometry. Appl Environ Microbiol 61:2506-13.

19. Lepère C, Domaizon I, Debroas D. 2008. Unexpected importance of potential parasites in the composition of the freshwater small-eukaryote community. Appl Environ Microbiol 74:2940-9.

20. Metfies K, Medlin L. 2007. Refining cryptophyte identification with DNA-microarrays. J Plankton Res 12:1071-1075.

21. Piwosz K, Kownacka J, Ameryk A, Zalewski M, Pernthaler J. 2016. Phenology of cryptomonads and the CRY1 lineage in a coastal brackish lagoon (Vistula Lagoon, Baltic Sea). J Phycol 52:626–637

22. Not F, Latasa M, Marie D, Cariou T, Vaulot D, Simon N. 2004. A single species, Micromonas pusilla (Prasinophyceae), dominates the eukaryotic picoplankton in the Western English Channel. Appl Environ Microbiol 70:4064-4072.

23. Grujčić V, Nuy JK, Salcher MM, Shabarova T, Kasalický V, Boenigk J, Jensen M, Šimek K. 2018. Cryptophyta as major bacterivores in freshwater summer plankton. The ISME Journal 12:1668–1681.

24. Medlin LK, Strieben S. 2010. Refining cryptophyte identification: matching cell fixation methods to FISH hybridisation of cryptomonads. J Appl Phycol 22:725-731.

25. Kahn P, Herfort L, Peterson TD, Zuber P. 2014. Discovery of a Katablepharis sp in the Columbia River estuary that is abundant during the spring and bears a unique large ribosomal subunit sequence element. Microbiologyopen 3:764-776.

26. Eller G, Toebe K, Medlin LK. 2007. Hierarchical probes at various taxonomic levels in the Haptophyta and a new division level probe for the Heterokonta. J Plankton Res 29:629-640.

27. Piwosz K, Spich K, Całkiewicz J, Weydmann A, Kubiszyn AM, Wiktor JM. 2015. Distribution of small phytoflagellates along an Arctic fjord transect. Environ Microbiol 17:2393–2406.

28. Zingone A, Chretiennot-Dinet MJ, Lange M, Medlin L. 1999. Morphological and genetic characterization of Phaeocystis cordata and P. jahnii (Prymnesiophyceae), two new species from the Mediterranean Sea. J Phycol 35:1322-1337.

29. Piwosz K. 2019. Weekly dynamics of abundance and size structure of specific nanophytoplankton lineages in coastal waters (Baltic Sea). Limnol Oceanogr 64:2172-2186.

30. Fried J, Ludwig W, Psenner R, Schleifer KH. 2002. Improvement of ciliate identification and quantification: a new protocol for fluorescence in situ hybridization (FISH) in combination with silver stain techniques. Syst Appl Microbiol 25:555-571.

31. Zhan Z, Stoeck T, Dunthorn M, Xu K. 2014. Identification of the pathogenic ciliate Pseudocohnilembus persalinus (Oligohymenophorea: Scuticociliatia) by fluorescence in situ hybridization. Eur J Protistol 50:16-24.

32. Gimmler A, Stoeck T. 2015. Mining environmental high-throughput sequence data sets to identify divergent amplicon clusters for phylogenetic reconstruction and morphotype visualization. Environmental Microbiology Reports 7:679-686.

33. del Campo J, Not F, Forn I, Sieracki ME, Massana R. 2013. Taming the smallest predators of the oceans. The ISME journal 7:351-358.

34. Piwosz K, Pernthaler J. 2011. Enrichment of omnivorous cercozoan nanoflagellates from coastal Baltic Sea waters. PLoS ONE 6:e24415.

35. Šimek K, Grujčić V, Mukherjee I, Kasalický V, Nedoma J, Posch T, Mehrshad M, Salcher MM. 2020. Cascading effects in freshwater microbial food webs by predatory Cercozoa, Katablepharidacea and ciliates feeding on aplastidic bacterivorous cryptophytes. FEMS Microbiol Ecol In press.

36. Massana R, del Campo J, Dinter C, Sommaruga R. 2007. Crash of a population of the marine heterotrophic flagellate Cafeteria roenbergensis by viral infection. Environ Microbiol 9:2660-2669.

37. Massana R, Terrado R, Forn I, Lovejoy C, Pedros-Alio C. 2006. Distribution and abundance of uncultured heterotrophic flagellates in the world oceans. Environ Microbiol 8:1515-1522.

38. Massana R, Guillou L, Diez B, Pedros-Alio C. 2002. Unveiling the Organisms behind Novel Eukaryotic Ribosomal DNA Sequences from the Ocean. Appl Environ Microbiol 68:4554-4558.

39. Giner CR, Forn I, Romac S, Logares R, de Vargas C, Massana R. 2016. Environmental sequencing provides reasonable estimates of the relative abundance of specific picoeukaryotes. Appl Environ Microbiol 82:4757-4766.

40. Kolodziej K, Stoeck T. 2007. Cellular identification of a novel uncultured marine stramenopile (MAST-12 clade) small-subunit rRNA gene sequence from a Norwegian estuary by use of fluorescence in situ hybridization-scanning electron microscopy. Appl Environ Microbiol 73:2718-2726.

41. Guillou L, Moon-van der Staay S-Y, Claustre H, Partensky F, Vaulot D. 1999. Diversity and abundance of Bolidophyceae (Heterokonta) in two oceanic regions. Appl Environ Microbiol 65:4528-4536.

42. Not F, Simon N, Biegala IC, Vaulot D. 2002. Application of fluorescent in situ hybridization coupled with tyramide signal amplification (FISH-TSA) to assess eukaryotic picoplankton composition. Aquat Microb Ecol 28:157-166.

43. Caron DA, Lim EL, Dennett MR, Gast RJ, Kosman C, DeLong EF. 1999. Molecular phylogenetic analysis of the heterotrophic Chrysophyte genus Paraphysomonas (Chrysophyceae), and the design of rRNA-targeted oligonucleotide probes for two species. J Phycol 35:824-837.

44. Lim EL, Dennett MR, Caron DA. 1999. The ecology of Paraphysomonas imperforata based on studies employing oligonucleotide probe identification in coastal water samples and enrichment cultures. Limnol Oceanogr 44:37-51.

45. Beardsley C, Knittel K, Amann R, Pernthaler J. 2005. Quantification and distinction of aplastidic and plastidic marine nanoplankton by fluorescence in situ hybridization. Aquat Microb Ecol 41:163-169.

46. Fuller NJ, Tarran GA, Cummings DG, Woodward EMS, Orcutt KM, Yallop M, Le Gall F, Scanlan DJ. 2006. Molecular analysis of photosynthetic picoeukaryote community structure along an Arabian Sea transect. Limnol Oceanogr 51:2502-2514.
