# Supplementary material for: CARD-FISH in the Sequencing Era: Opening a New Universe of Protistan Ecology
Source: Front Microbiol. 2021 Mar 4;12:640066. doi: 10.3389/fmicb.2021.640066 (PMC7970053; doi:10.3389/fmicb.2021.640066)
Supplement: Supplementary Table 2 — Volumes of formamide (form.) and water for 20 ml of hybridization buffer (HB), volumes of 5 M NaCl in 50 ml of washing buffer at 37°C (for hybridization at 35°C) and 48°C (for hybridization at 46°C). The recipes for HB and WB can be found in Supplementary File 4. [file Table_2.PDF]

Supplementary Table S2. Volumes of formamide (form.) and water for 20 ml of hybridization buffer (HB), volumes of 5 M NaCl in 50 ml of washing buffer at 37°C (for hybridization at 35°C) and 48°C (for hybridization at 46°C). The recipes for HB and WB can be found in Supplementary File 4.

% form. in HB	HB (20 mL)		WB (50 mL)			
			Washing at 37°C		Washing at 48°C	
	mL form.	mL DI water	Concn. NaCl (mol L^{-1})	µL of 5 M NaCl	Concn. NaCl (mol L^{-1})	µL of 5 M NaCl
**0**	0	14	--	--	0.900	8900
**5**	1	13	--	--	0.636	6260
**10**	2	12	--	--	0.450	4400
**15**	3	11	--	--	0.318	3080
**20**	4	10	0.145	1350	0.225	2150
**25**	5	9	0.105	950	0.159	1490
**30**	6	8	0.074	640	0.112	1020
**35**	7	7	0.052	420	0.080	700
**40**	8	6	0.037	270	0.056	460
**45**	9	5	0.026	160	0.040	300
**50**	10	4	0.019	90	0.028	180
**55**	11	3	0.013	30	0.020	100
**60**	12	2	0.009	0	0.014	40
**65**	13	1	0.007	0	--	--
**70**	14	0	0.005	0	--	--
