# Supplementary material for: CARD-FISH in the Sequencing Era: Opening a New Universe of Protistan Ecology
Source: Front Microbiol. 2021 Mar 4;12:640066. doi: 10.3389/fmicb.2021.640066 (PMC7970053; doi:10.3389/fmicb.2021.640066)
Supplement: Supplementary Table 3 — Composition of buffers according to two most commonly used protocols: Not et al. (2002) and Piwosz and Pernthaler (2010). Concentrations of formamide in the hybridization buffer need to be optimized for each probe. The detailed instructions on how to prepare the buffers are given in Supplementary File 4. [file Table_3.PDF]

Supplementary Table S3. Composition of buffers according to two most commonly used protocols: Piwosz and Pernthaler (2010) and Not et al. (2002). Concentrations of formamide in the hybridization buffer need to be optimized for each probe. The detail instructions how to prepare the buffers are given in Supplementary File 4.

**Hybridization buffer**	**Reference**		**Amplification buffer**	**Reference**
**Reagent**	Not et al., 2002	Piwosz and Pernthaler, 2010	Reagent	Piwosz and Pernthaler, 2010
**NaCl**	0.9 M	0.9 M	NaCl	2 M
**Tris-HCl (pH 7.4-7.5)**	20 mM	20 mM	PBS	1x
**Dextran sulfate (w/v)**	0	10%	Dextran sulfate (w/v)	10%
**Blocking agent (w/v)**	10%	1%	Blocking agent (w/v)	0.1%
**probe**	1:10	1:100	Tyramides	1:500-1:1000
**SDS (v/v)**	0.01%	0.01%	0	0
**formamide**	Depends on probe		--	--

References:

Not, F., Simon, N., Biegala, I.C., and Vaulot, D. (2002). Application of fluorescent in situ hybridization coupled with tyramide signal amplification (FISH-TSA) to assess eukaryotic picoplankton composition. *Aquatic Microbial Ecology* 28**,** 157-166.

Piwosz, K., and Pernthaler, J. (2010). Seasonal population dynamics and trophic role of planktonic nanoflagellates in coastal surface waters of the Southern Baltic Sea. *Environmental Microbiology* 12**,** 364-377.
